# ANXA2‐mediated Phagocytosis Generates AR^+^ Macrophages to Confer Enzalutamide Resistance in Prostate Cancer

**DOI:** 10.1002/advs.75290

**Published:** 2026-04-16

**Authors:** Yong Luo, Tianlong Luo, Lingfeng Li, Qi Sun, Dongquan Li, Shanhe Huang, Zonglin Li, Weilong Lin, Yuan Ou, Tao Du, Shengmeng Peng, Kewei Xu, Bisheng Cheng, Hai Huang

**Affiliations:** ^1^ Department of Urology Sun Yat‐sen Memorial Hospital Sun Yat‐sen University Guangzhou Guangdong China; ^2^ Guangdong Provincial Key Laboratory of Malignant Tumor Epigenetics and Gene Regulation Sun Yat‐Sen Memorial Hospital Sun Yat‐Sen University Guangzhou Guangdong China; ^3^ Vancouver Prostate Centre Department of Urologic Sciences University of British Columbia Vancouver British Columbia Canada; ^4^ Beijing Hospital National Center of Gerontology Institute of Geriatric Medicine Chinese Academy of Medical Sciences & Peking Union Medical College Beijing China; ^5^ Department of Urology Nanfang Hospital Southern Medical University Guangzhou Guangdong China; ^6^ Department of Obstetrics and Gynecology Sun Yat‐Sen Memorial Hospital Sun Yat‐Sen University Guangzhou Guangdong China; ^7^ Guangdong Provincial Clinical Research Center for Urological Diseases Sun Yat‐Sen Memorial Hospital Sun Yat‐Sen University Guangzhou Guangdong China; ^8^ Southern Medical University Guangzhou Guangdong China; ^9^ Department of Clinical Oncology Li Ka Shing Faculty of Medicine The University of Hong Kong Hong Kong SAR China; ^10^ Department of Clinical Oncology The University of Hong Kong‐Shenzhen Hospital Shenzhen China; ^11^ Department of Surgery Division of Urology Beth Israel Deaconess Medical Center Harvard Medical School Boston Massachusetts USA; ^12^ Department of Urology The Sixth Affiliated Hospital of Guangzhou Medical University Qingyuan People's Hospital Qingyuan Guangdong China

**Keywords:** AR^+^ TAMs, CRPC, enzalutamide resistance, IL‐6 signaling, phagocytosis

## Abstract

Resistance to second‐generation antiandrogens like enzalutamide (ENZ) in castration‐resistant prostate cancer (CRPC) is a major clinical challenge, yet the role in the tumor microenvironment remains poorly understood. This study identifies a unique AR‐positive tumor‐associated macrophages (AR^+^ TAMs) subpopulation, enriched in ENZ‐resistant patients and correlated with poor prognosis, which acquires functional AR protein not through endogenous expression but via ANXA2‐dependent phagocytosis of tumor cells. The internalized AR protein translocates to the macrophage nucleus, directly binds the IL‐6 promoter to enhance its transcription and secretion. Macrophage‐derived IL‐6 subsequently activates the JAK2/STAT3 pathway in cancer cells, suppressing ENZ‐induced apoptosis and conferring therapeutic resistance. Genetic or pharmacological blockade of IL‐6 signaling restored ENZ sensitivity in vitro and in vivo, and combining an anti‐IL‐6 antibody with ENZ synergistically overcomes resistance in patient‐derived xenograft and orthotopic models. These findings reveal a novel phagocytosis‐mediated, paracrine mechanism of ENZ resistance orchestrated by AR^+^ TAMs, challenging the tumor‐centric view of therapy failure and providing a strong rationale for co‐targeting the IL‐6 pathway to improve outcomes of AR‐directed therapy in CRPC.

## Graphical abstract

A novel resistance mechanism is mediated through phagocytosis of cancer cells by AR^+^ TAMs. This process, dependent on ANXA2, enables macrophages to acquire AR protein from engulfed tumor cells. The internalized AR translocates into the macrophage nucleus, where it binds directly to the IL‐6 promoter, augmenting IL‐6 transcription and secretion. Subsequently, macrophage‐derived IL‐6 activates the IL‐6R on cancer cells, triggering JAK2/STAT3 signaling that suppresses ENZ‐induced apoptosis and confers therapeutic resistance. Importantly, this resistance axis can be effectively disrupted by anti‐IL‐6 antibody treatment, which restores ENZ sensitivity.

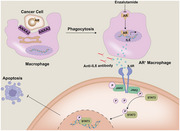

## 1 Introduction

The transition from locally advanced prostate cancer (PCa) to castration‐resistant PCa (CRPC) and the emergence of treatment refractoriness represent the central challenge in clinical management [1]. Despite the initial clinical benefits of second‐generation antiandrogen therapy (SGAT) drugs like enzalutamide (ENZ), resistance almost invariably develops. This highlights the shortcomings of the current focus on cancer cells as the sole cause of treatment failure [2, 3]. Beyond established cell‐intrinsic mechanisms like androgen receptor (AR) amplification, mutation, and AR‐V7 expression, emerging evidence implicates the tumor microenvironment (TME) as a key sanctuary and active contributor to treatment resistance [4, 5]. The dynamic interplay between tumor cells and their stromal milieu promotes resistance pathways that are not targeted by existing standard therapies, thereby underscoring the imperative to define these extrinsic mechanisms.

Beyond its well‐established role as a transcriptional master regulator in prostate epithelium, AR has an extended functional role within the stromal compartment [6]. Evidence indicates that AR signaling in cancer‐associated fibroblasts (CAFs) can exert a more profound tumor‐promoting effect than epithelial AR in early tumorigenesis [7]. Furthermore, the loss of AR in CAFs initiates a transcriptional reprogramming, thereby driving the progression of non‐prostate malignancies [8, 9]. Notably, AR signaling extends beyond stromal cells and represents a key regulatory mechanism in the myeloid compartment, especially within tumor‐associated macrophages (TAMs), thereby linking tumor cells with immune responses [10].

Within the prostate TME, TAMs represent one of the predominant immune cell populations and are well‐established orchestrators of tumor progression and therapy resistance [11]. Their impact on the immunological landscape is profound and multifunctional [12, 13]. TAMs suppress adaptive antitumor immunity through several distinct pathways: secretion of immunosuppressive cytokines, including IL ‐ 10 and TGF‐β [14]; expression of immune checkpoint ligands such as PD‐L1 to directly inhibit cytotoxic T cells [15]; recruitment of regulatory T cells via chemokine production [16]; and metabolic disruption of T cells function by depleting essential nutrients like L‐arginine and generating reactive oxygen species [17]. By fostering an immunosuppressive niche, TAMs not only promote immune evasion but also indirectly enhance tumor cell survival under therapeutic stress, thereby positioning TAMs as a pivotal therapeutic target in CRPC [18]. The functional contributions of TAMs are largely mediated by their extensive secretome [14, 19]. Cytokines represent the primary mediators of macrophage‐tumor cell communication, with interleukin‐6 (IL‐6) playing a central role in PCa pathogenesis [20]. IL‐6 signals via the IL‐6 receptor and activates the JAK2/STAT3 pathway, thereby promoting tumor cell proliferation and stem‐like properties [21, 22]. Importantly, a well‐characterized bidirectional cross‐talk connects IL‐6 and AR signaling, offering a mechanistic basis for how inflammatory cytokines can directly contribute to resistance against AR‐directed therapy.

The identity heterogeneity of TAMs is governed by the integration of extrinsic signals and intrinsic cellular capacities [23]. TAMs identity is critically shaped by core biological processes, with phagocytosis representing a fundamental mechanism [24]. This highly sophisticated cellular function allows macrophages to engulf and internalize large particles such as apoptotic cells, pathogens, and even viable tumor cells [25]. Phagocytosis is not simply a scavenging activity but an immunologically active process that entails coordinated engagement of receptors, including Fcγ receptors, complement receptors, and scavenger receptors, together with extensive actin cytoskeleton reorganization and intricate membrane trafficking [26, 27]. The molecular apparatus that regulates this process, particularly in the context of tumor cell engulfment, remains incompletely understood.

ANXA2, a membrane‐associated protein recognized for its role in actin dynamics, membrane organization, and vesicle fusion in other systems, emerges as a compelling yet unvalidated candidate that may mediate the targeted phagocytic clearance of prostate tumor cells [28]. Importantly, the phagocytic uptake of cellular material can act as a potent stimulus that reprograms macrophage phenotype and function, potentially generating novel and functionally distinct TAMs subsets within the TME [29]. The concept that a distinct TAMs subset acts as its dominant source, and that this production is directly regulated by a transcription factor acquired via phagocytosis, would represent a critical advance in understanding CRPC pathophysiology.

Here, we identify a distinct and clinically relevant subpopulation of AR‐positive TAMs (AR^+^ TAMs) that mediates ENZ resistance and predicts poor prognosis in CRPC. These macrophages acquire AR protein not through canonical expression, but via ANXA2‐dependent phagocytosis, revealing a previously unrecognized origin for this pivotal macrophage subset. Furthermore, we demonstrate that the acquired AR in macrophages binds directly to the IL‐6 promoter, driving its transcription and subsequent secretion. The resulting macrophage‐derived IL‐6 establishes a critical paracrine circuit that activates the JAK2/STAT3 pro‐survival pathway in tumor cells, thereby attenuating ENZ‐induced apoptosis. Preclinical studies comprehensively validate that therapeutic inhibition of this macrophage‐driven IL‐6/JAK2/STAT3 axis synergizes with ENZ to overcome resistance and suppress tumor growth. These findings propose a novel and mechanistically rationalized combination strategy for the treatment of CRPC.

## 2 Results

### 2.1 Identification of AR^+^ TAMs as a Mediator of Resistance and Poor Prognosis in CRPC

**FIGURE 1:**
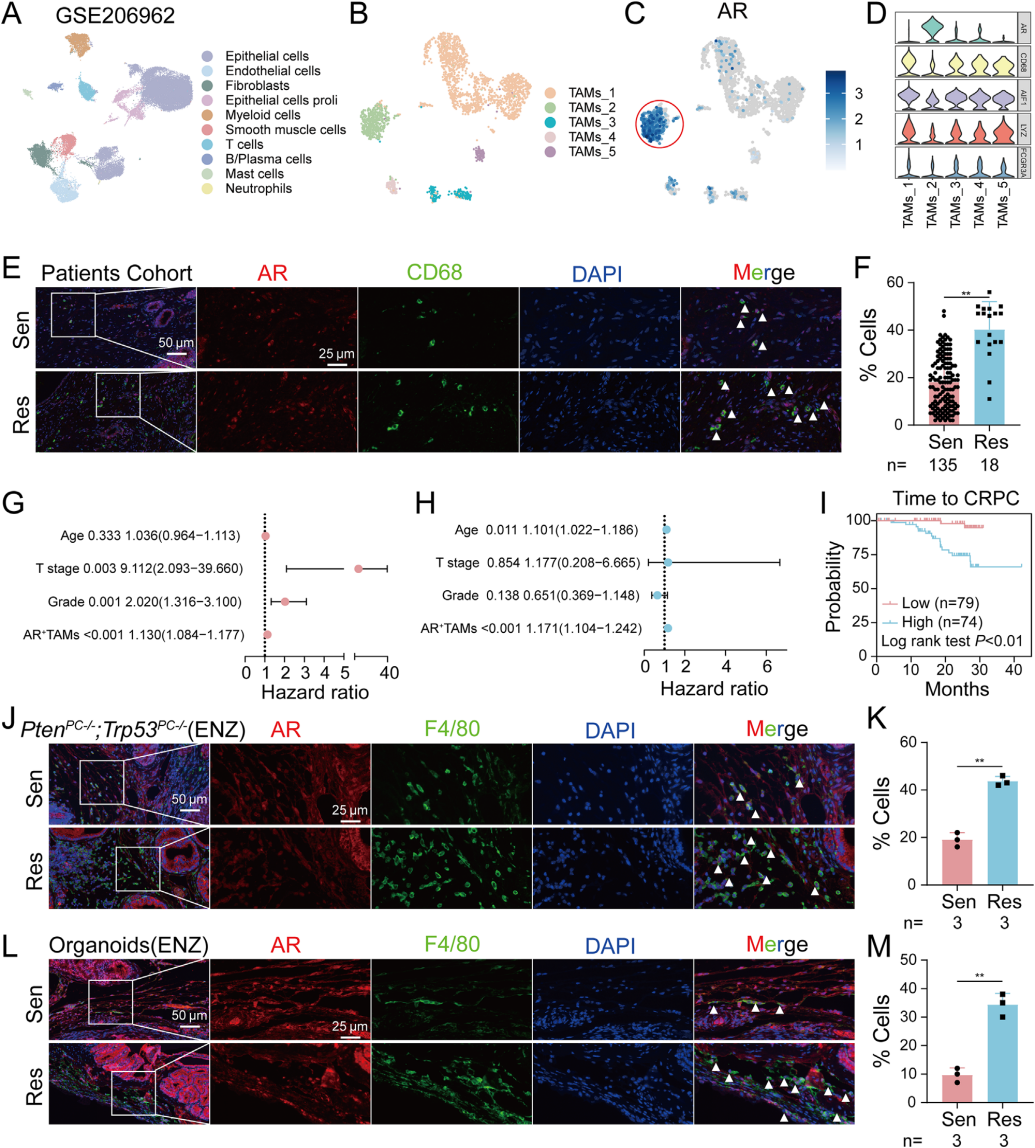
Identification of AR^+^ TAMs as mediators of resistance and poor prognosis in CRPC. (A) UMAP plot showing major cell types in GSE206962 from ENZ‐resistant PCa. (B) UMAP plot illustrating five distinct TAMs subpopulations (TAMs_1‐TAMs_5). (C) UMAP plot depicting AR^+^ TAMs subpopulation. (D) Violin plots displaying the AR expression levels across different TAMs subpopulations. (E) Representative mIF images of tumor tissues from SGAT‐sensitive (Sen) and resistant (Res) PCa patients. Sections were co‐stained for AR (red), CD68 (green), and DAPI (blue). White arrowheads in the merged panels indicate AR^+^ TAMs. Scale bars, 50 µm (low‐magnification images) and 25 µm (high‐magnification insets). (F) Quantification of AR^+^ TAMs as a percentage of the total macrophage population (CD68^+^) in the indicated patients cohort. (G, H) Forest plots showing the hazard ratios for CRPC‐free survival based on univariate (G) and multivariate (H) Cox regression analyses, incorporating AR^+^ TAMs, age, T stage, and tumor grade as variables. (I) Kaplan–Meier curves for CRPC‐free survival in PCa patients stratified by AR^+^ TAMs infiltration levels. (J) Representative mIF images of tumor tissues from *Pten/Trp53* double‐knockout mice with ENZ‐sensitive (Sen) or resistant (Res) PCa. Tissues were stained for AR (red), F4/80 (green), and DAPI (blue), with white arrowheads indicating AR^+^ TAMs. Scale bars, 50 and 25 µm (insets). (K) Quantification of the AR^+^ TAMs among total F4/80^+^ macrophages in the murine tumors. (L) Representative mIF images of tumor organoids derived from *Pten/Trp53*‐deficient cells under ENZ‐sensitive (Sen) or resistant (Res) conditions. Fluorescence staining and annotations are identical to (J). Scale bars, 50 and 25 µm (insets). (M) Quantification of the AR^+^ TAMs percentage within the F4/80^+^ population in the organoid models.

While the AR is predominantly expressed in prostate epithelial cells, its expression has also been documented in immune cells and other stromal components [30]. Clustering analysis of single‐cell RNA sequencing (scRNA‐seq) data from ENZ‐resistant PCa (GSE206962) revealed a distinct macrophage subpopulation positive for AR expression (Figure 1A–D; Figure S1E–G). This finding was validated in an independent scRNA‐seq dataset from patients with CRPC (GSE137829, Figure S1A–D,H,I). Using multiplex immunofluorescence (mIF) staining on a clinical cohort of 153 PCa patients, including SGAT‐resistant cases, we confirmed the presence of AR^+^ TAMs in the prostate TME. Notably, the proportion of AR^+^ cells within the total TAMs population was significantly elevated in SGAT‐resistant patients (Figure 1E,F). Furthermore, a higher AR^+^ TAM density correlated with advanced clinical stage, higher tumor grade, and increased serum PSA levels (Figure S1J–O). Both univariate and multivariate Cox regression analyses identified AR^+^ TAMs as an independent predictor of shorter time to CRPC (Figure 1G,H). Consistently, Kaplan–Meier survival analysis showed that patients with higher AR^+^ TAMs infiltration experienced significantly shorter CRPC‐free survival (Figure 1I). To validate these observations, we employed a *Pten/Trp53* double‐knockout mouse model and an orthotopic tumor model derived from *Pten/Trp53*‐deficient organoids. Both models were subjected to castration and ENZ treatment to induce an ENZ‐resistant state. mIF analysis of tumor tissues from two models confirmed the presence of AR^+^ TAMs. In line with the above results, the ENZ‐resistant mouse models exhibited a significantly increased proportion of AR^+^ cells within the total TAMs population compared to controls (Figure 1J–M).

### 2.2 ANXA2‐Dependent Phagocytosis Drives the Generation of AR^+^ TAMs

**FIGURE 2:**
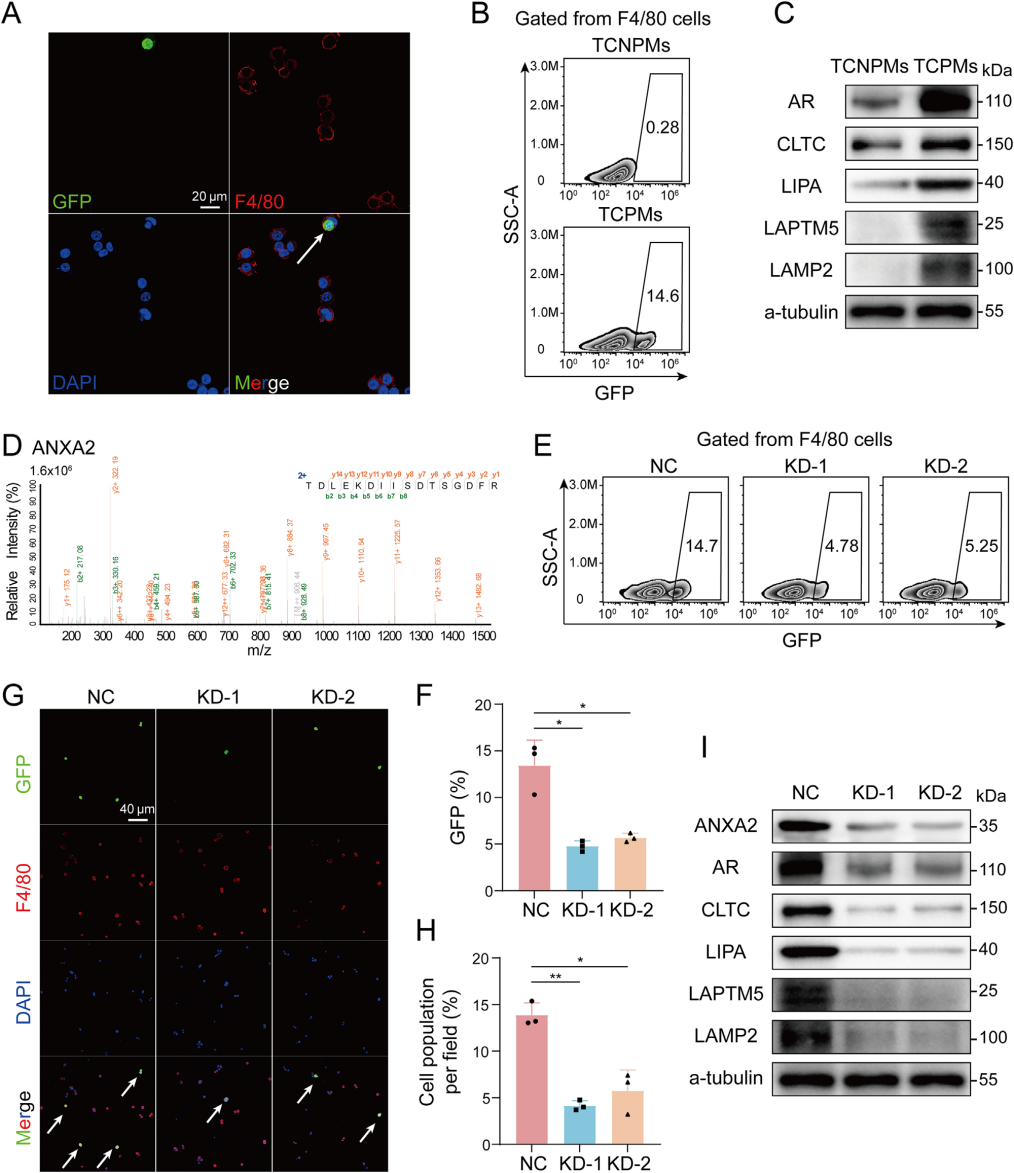
ANXA2‐dependent phagocytosis drives the generation of AR^+^ TAMs. (A) Representative confocal microscopy images showing the uptake of GFP‐labeled tumor cells by macrophages (stained with F4/80, red; nuclei stained with DAPI, blue). White arrows indicate engulfed cells. Scale bar = 20 µm. (B) Representative flow cytometry plots identifying GFP^+^ cells gated from F4/80^+^ macrophages, defining the TCNPMs and TCPMs. (C) WB analysis evaluating the protein levels of AR, CLTC, LIPA, LAPTM5, and LAMP2 in TCNPMs and TCPMs. (D) Mass spectrometry spectrum identifying ANXA2. (E) Flow cytometry analysis of GFP^+^ cells within the F4/80^+^ gate following ANXA2 knockdown (KD‐1 and KD‐2) versus negative control (NC). (F) Quantification of the percentage of GFP^+^ cells from (E). (G) Confocal microscopy images visualizing macrophage phagocytic capacity in NC and ANXA2‐deficient groups. Staining included GFP (green), F4/80 (red), and DAPI (blue), with white arrows pointing to internalized cells. Scale bar = 40 µm. (H) Quantification of phagocytosing macrophage populations per microscopic field. (I) WB analysis of ANXA2, AR, and phagocytosis‐associated proteins (CLTC, LIPA, LAPTM5, LAMP2) in macrophages subjected to ANXA2 knockdown.

Given the predominant expression of AR in prostate epithelial cells and its minimal presence in macrophages, together with the robust phagocytic capacity of the latter, we aimed to investigate the origin of AR^+^ TAMs. To visualize phagocytosis, murine PCa cell line RM1 cells were transduced with GFP, enabling detection of internalized tumor cells by both confocal microscopy and FACS. A co‐culture system of macrophages and tumor cells was established. Macrophages were labeled using F4/80, and confocal microscopy clearly captured macrophages engulfing tumor cells (Figure 2A), a finding further confirmed by FACS analysis (Figure 2B). Tumor cell‐phagocytosing macrophages (TCPMs) and tumor cell‐nonphagocytosing macrophages (TCNPMs) populations were then isolated through FACS (Figure 2B). Western blot (WB) analysis revealed significantly elevated AR expression in TCPMs. Moreover, this subset exhibited markedly increased protein levels of key regulators of lysosomal and phagocytic signaling pathways, including clathrin heavy chain (CLTC), lipase A, lysosomal acid lipase A (LIPA), lysosomal protein transmembrane 5 (LAPTM5), and lysosomal‐associated membrane protein 2 (LAMP2) (Figure 2C). Since phagocytosis involves dynamic membrane remodeling, we performed membrane protein‐focused mass spectrometry on sorted macrophages (Figure S2A), which identified ANXA2 as a prominent candidate (Figure 2D). We found that ANXA2 expression was positively correlated with macrophage markers and phagocytosis‐related markers such as CD68, CD163, CD206, CD209, and DECTIN‐1 (Figure S2B–F). ANXA2 knockdown significantly attenuated macrophage‐mediated phagocytosis of tumor cells, as assessed by flow cytometry (Figure 2E,F) and confocal microscopy (Figure 2G,H). Furthermore, ANXA2 silencing downregulated AR expression and key phagocytosis‐related proteins, as confirmed by WB (Figure 2I).

### 2.3 AR^+^ TAMs Suppress Tumor Cell Apoptosis via Paracrine IL‐6/IL‐6R Signaling

**FIGURE 3:**
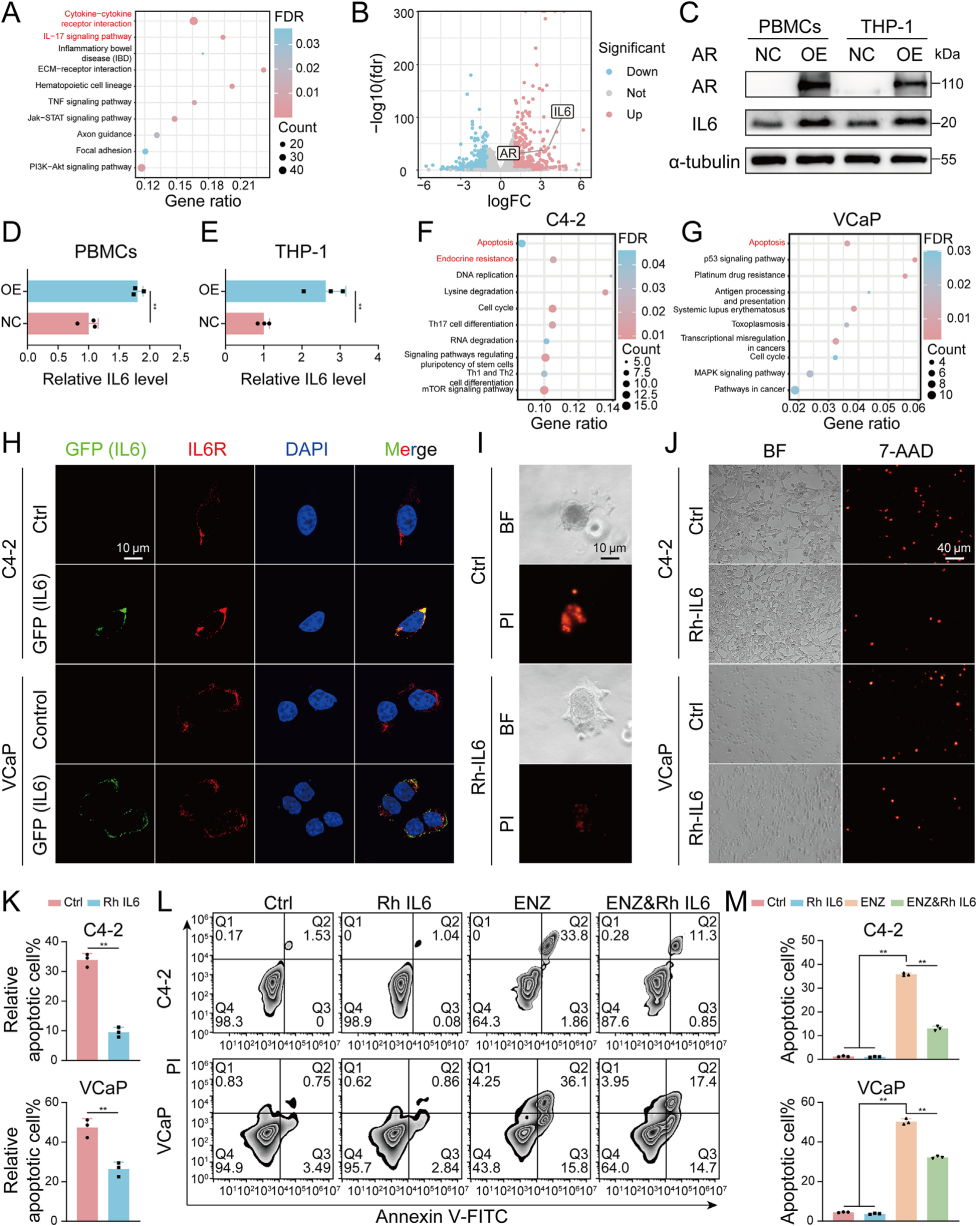
AR^+^ TAMs suppress tumor cell apoptosis via paracrine IL‐6/IL‐6R signaling. (A) KEGG pathway enrichment analysis of differentially expressed genes (DEGs) in THP‐1 cells following AR overexpression (OE). (B) Volcano plot visualizing DEGs in AR‐overexpressing THP‐1 cells, with AR and IL‐6 explicitly highlighted. (C) WB evaluating AR and IL‐6 protein levels in PBMCs and THP‐1 cells transfected with AR OE plasmid or negative control (NC) vector. (D, E) ELISA quantification of secreted IL‐6 levels in the culture supernatants of PBMCs (D) and THP‐1 cells (E) subjected to AR OE or NC. (F, G) KEGG pathway enrichment analysis of DEGs in C4‐2 (F) and VCaP (G) cells treated with rhIL‐6. (H) Representative confocal immunofluorescence images demonstrating the co‐localization (yellow) of macrophage‐derived GFP‐IL‐6 (green) with the IL‐6R (red) on C4‐2 and VCaP cells. Scale bar = 10um. (I) Representative images of PI staining in *Pten/p53* double‐knockout organoids treated with rhIL‐6 or vehicle control under ENZ treatment. Scale bar = 10um. (J) Representative images of 7‐AAD staining in C4‐2 and VCaP cells co‐treated with ENZ and either rhIL‐6 or control. Scale bar = 40um. (K) Quantification of the relative percentage of apoptotic cells from (J). (L, M) Representative flow cytometric plots (L) and quantification (M) of apoptosis in C4‐2 and VCaP cells following the indicated treatments (Ctrl, rhIL‐6, ENZ, or ENZ + rhIL‐6).

Cytokines are pivotal in shaping the tumor immune microenvironment, influencing cancer progression and therapeutic resistance [31]. To explore the contribution of AR^+^ TAMs to ENZ resistance, we performed transcriptomic profiling of THP‐1 following AR overexpression. AR^+^ TAMs profoundly remodeled the cytokine expression profile, with significant enrichments in pathways such as “cytokine‐cytokine receptor interaction” and the “IL‐17 signaling pathway” (Figure 3A). Comprehensive profiling of an extended panel of TAMs‐associated secretory factors revealed that IL‐6 exhibited the most profound upregulation among all differentially expressed cytokines (Figure 3B; Figure S3A,B). Consistent with transcriptomic findings, AR overexpression in both PBMCs and THP‐1 cells led to substantial increases in IL‐6 at the mRNA (Figure S3C,D) and protein levels (Figure 3C), along with elevated IL‐6 in the culture supernatant (Figure 3D,E). Conversely, AR knockdown significantly reduced IL‐6 expression intracellularly (Figure S3E) and in conditioned media (Figure S3F,G). To investigate whether AR^+^ TAMs confer therapy resistance via IL‐6 signaling, we treated tumor cells with recombinant human IL‐6 (rhIL‐6) and performed RNA sequencing. Pathway analysis revealed significant enrichment of gene sets associated with apoptosis and endocrine resistance (Figure 3F,G). To validate functional interaction, we co‐cultured tumor cells with macrophages transfected with a GFP‐tagged IL‐6 plasmid (Figure S3H). Immunofluorescence confirmed co‐localization of macrophage‐derived GFP‐IL‐6 with IL‐6 receptor (IL‐6R) on tumor cells, demonstrating direct ligand‐receptor engagement (Figure 3H). Functional validation using *Pten/p53* double‐knockout organoids showed that rhIL‐6 effectively rescued ENZ‐induced cell apoptosis, as evidenced by reduced propidium iodide (PI, Figure 3I) and 7‐AAD staining (Figure 3J,K). Flow cytometric analysis further confirmed that IL‐6 significantly suppressed ENZ‐triggered apoptosis (Figure 3L,M).

### 2.4 AR^+^ TAMs Confer ENZ Resistance via IL‐6‐Mediated Anti‐Apoptotic Signaling

**FIGURE 4:**
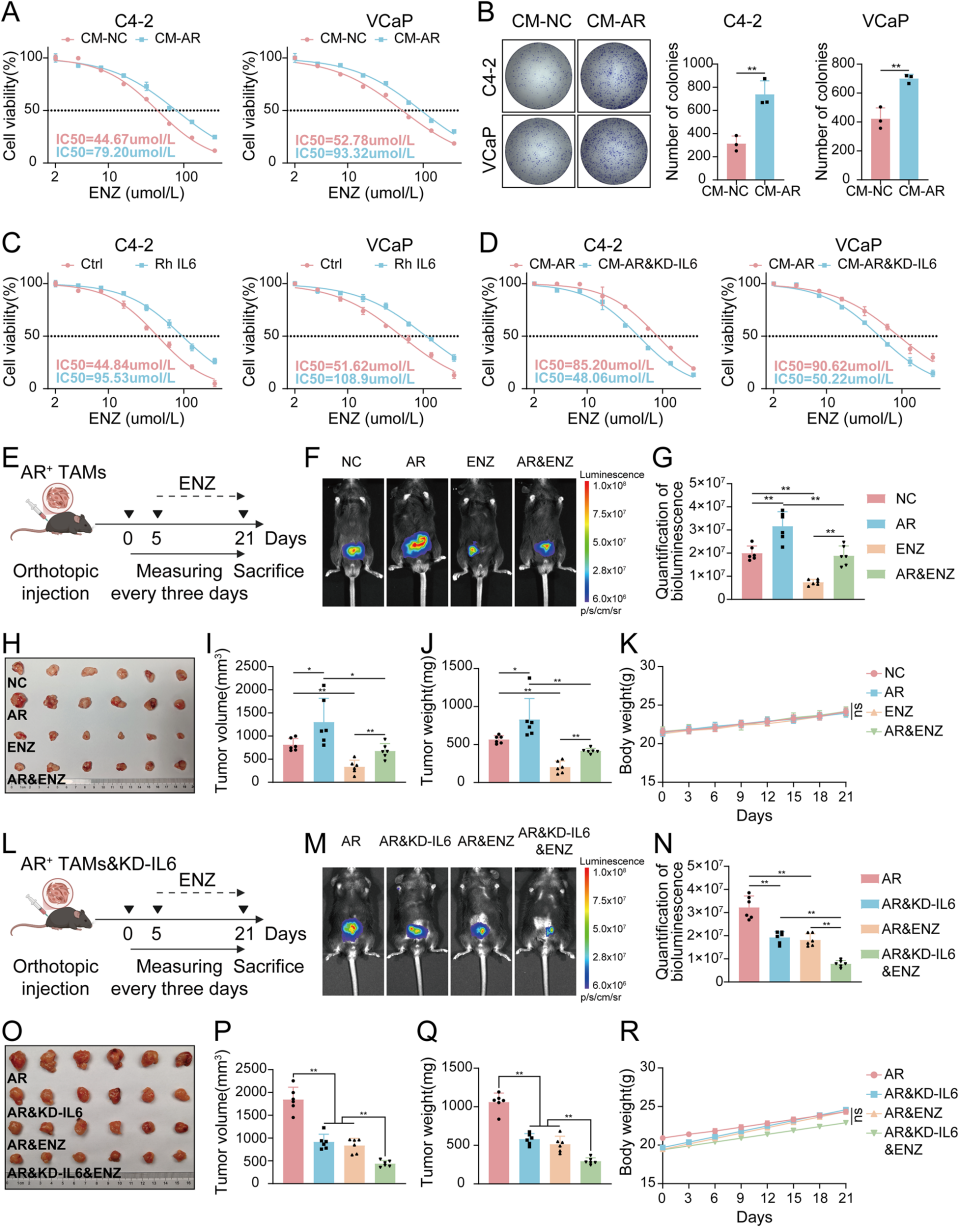
AR^+^ TAMs confer ENZ resistance via IL‐6‐mediated anti‐apoptotic signaling. (A) Dose‐response curves of cell viability in C4‐2 and VCaP cells treated with indicated concentrations of ENZ in the presence of CM from AR^+^ TAMs (CM‐AR) or control TAMs (CM‐NC). Calculated IC50 values are presented. (B) Representative images and quantitative analysis of colony formation assays for C4‐2 and VCaP cells cultured with CM‐AR or CM‐NC. (C) Cell viability curves of C4‐2 and VCaP cells treated with ENZ in combination with rhIL‐6 or vehicle control (Ctrl). (D) Cell viability of C4‐2 and VCaP cells treated with ENZ in the presence of CM from IL‐6‐silenced AR^+^ TAMs (CM‐AR&KD‐IL‐6) or control AR^+^ TAMs (CM‐AR). (E) Schematic illustration of the in vivo experimental design: Orthotopic co‐implantation of RM1 cells with AR^+^ TAMs, followed by ENZ treatment. (F–K) in vivo evaluation of the models described in (E), displaying representative bioluminescence images (F) and corresponding quantification (G), gross morphological images of excised tumors (H), terminal tumor volumes (I) and weights (J), and monitored murine body weights (K). (L) Schematic of the parallel in vivo experiment utilizing AR^+^ TAMs with IL‐6 knockdown prior to ENZ treatment. (M–R) Corresponding in vivo tumor progression metrics for the cohort in (L), including bioluminescence imaging (M, N), macroscopic tumor morphology (O), terminal tumor volumes (P) and weights (Q), and systemic body weights trajectories (R).

To investigate whether AR^+^ TAMs drive therapeutic failure, PCa cells cultured with conditioned medium (CM) from AR^+^ TAMs exhibited increasing resistance to ENZ in a dose‐dependent manner (Figure 4A). This ENZ‐resistant phenotype was further supported by colony formation assays, which revealed a significant protective effect conferred by CM from AR^+^ TAMs (Figure 4B). In contrast, CM from AR‐knockdown TAMs enhanced tumor cell sensitivity to ENZ (Figure S4A). To explore the mechanism, we treated tumor cells with rhIL‐6 and found that IL‐6 alone was sufficient to induce ENZ resistance (Figure 4C). Consistently, CM from IL‐6‐knockdown TAMs increased cellular susceptibility to ENZ (Figures S3I and S4B). We then generated IL‐6‐deficient AR^+^ TAMs and demonstrated that their CM failed to promote ENZ resistance (Figure 4D). In vitro, using *Pten/p53* double‐knockout prostate organoids, the results showed that CM from IL‐6‐knockdown AR^+^ TAMs did not protect organoids from ENZ‐induced cell death (Figure S4C). Apoptosis analysis via 7‐AAD staining and flow cytometry further confirmed that loss of IL‐6 in AR^+^ TAMs abolished their capacity to suppress ENZ‐induced apoptosis (Figure S4D–G).

We next validated these findings in vivo. RM1 were co‐implanted orthotopically with AR^+^ TAMs. After 5 days, mice were treated with ENZ (Figure 4E). In vivo bioluminescence imaging, macroscopic tumor examination, tumor volumes, and tumor weights measurements collectively indicated that AR^+^ TAMs conferred significant resistance to ENZ (Figure 4F–J). Body weights did not differ significantly among the four experimental groups (Figure 4K). In a separate in vivo experiment, IL‐6 was knocked down in AR^+^ TAMs prior to co‐implantation with RM1 cells(Figure 4L). ENZ treatment following IL‐6 knockdown abrogated the resistance mediated by AR^+^ TAMs, as evidenced by bioluminescence imaging, gross pathology, tumor volumes, and tumor weights analysis (Figures 4M–Q). Animal body weights remained comparable across groups (Figure 4R). Immunohistochemical analysis (IHC) of tumors from both in vivo studies revealed that AR^+^ TAMs infiltration markedly increased Ki‐67 expression, which was attenuated upon IL‐6 knockdown. Cleaved caspase‐3 levels were significantly suppressed in AR^+^ TAMs‐containing tumors but were restored following IL‐6 knockdown in TAMs (Figure S4H–K).

### 2.5 AR^+^ TAMs Drive ENZ Resistance by Transcriptionally Activating IL‐6

**FIGURE 5:**
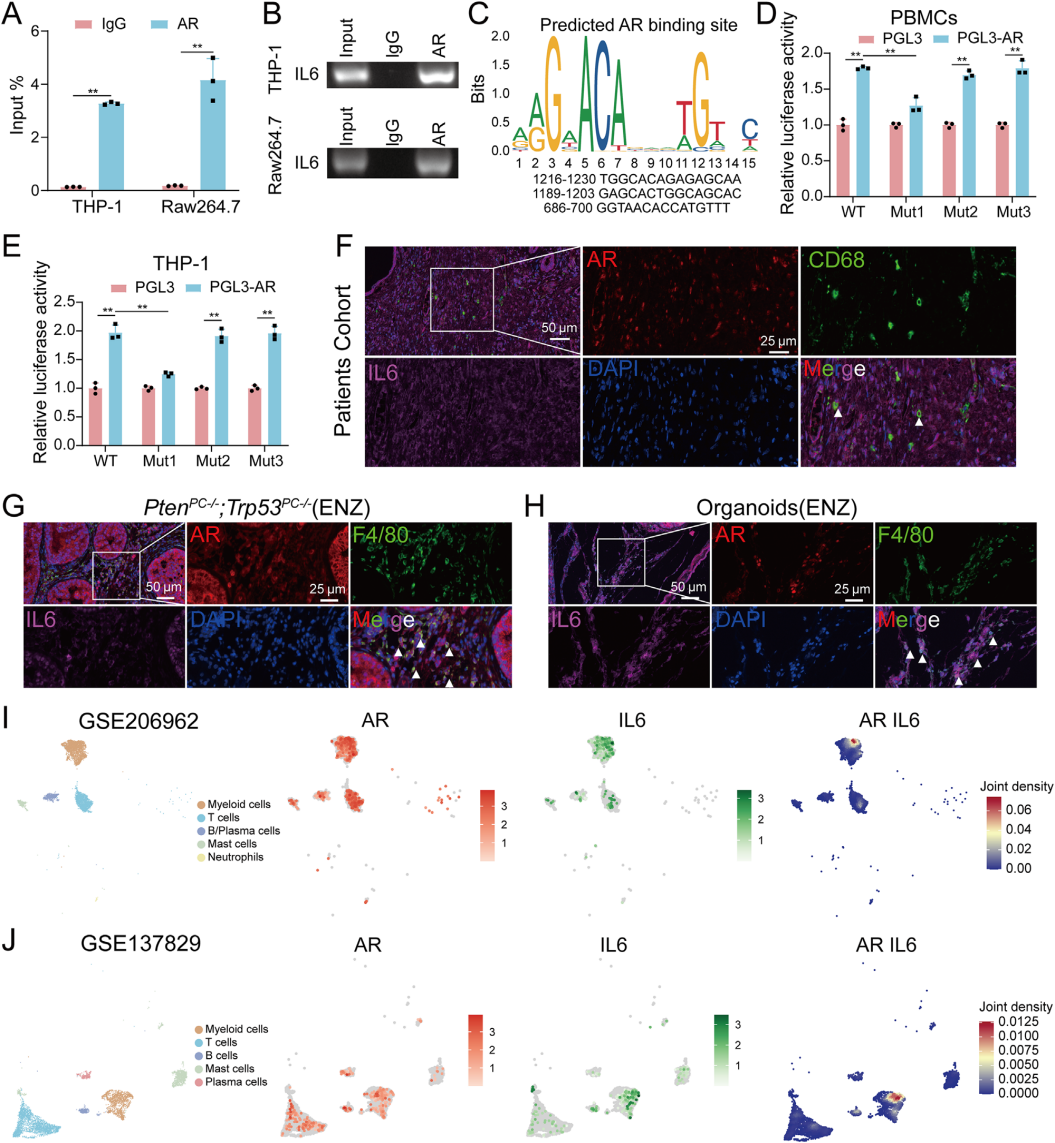
AR^+^ TAMs drive ENZ resistance by transcriptionally activating *IL‐6*. (A, B) ChIP‐qPCR (A) and semi‐quantitative PCR (B) analyses demonstrating AR enrichment at the *IL‐6* promoter in THP‐1 (human) and Raw264.7 (murine) macrophage cell lines. IgG was used as a negative control. (C) Sequence logo and positions of the predicted AR‐binding motifs within the *IL‐6* promoter, derived from the JASPAR database. (D, E) Dual‐luciferase reporter assays evaluating promoter activity in human PBMCs (D) and THP‐1 cells (E) co‐transfected with an AR‐overexpression plasmid and either wild‐type (WT) or mutated (Mut1, Mut2, Mut3) *IL‐6* promoter‐driven luciferase constructs. (F) Representative mIF staining for AR (red), CD68 (green), IL‐6 (purple), and DAPI (blue) in human CRPC tissue sections. White arrowheads indicate co‐localization of AR, CD68, and IL‐6. Scale bars, 50 µm (low magnification) and 25 µm (high‐magnification insets). (G, H) Representative mIF images of tumor tissues from ENZ‐resistant *Pten* ^PC‐/−^; *Trp53* ^PC‐/−^ mice (G) and corresponding tumor organoids (H). Sections were co‐stained for AR (red), F4/80 (green), IL‐6 (purple), and DAPI (blue). White arrowheads highlight AR^+^ F4/80^+^ IL‐6^+^ TAMs. Scale bars, 50 and 25 µm (insets). (I, J) UMAP and joint density plots generated from scRNA‐seq datasets GSE206962 (I) and GSE137829 (J), illustrating the robust co‐expression of *AR* and *IL‐6* transcripts specifically within TAMs.

Given the positive correlation between AR and IL‐6, both expressed in TAMs in PCa, we investigated the mechanism by which AR^+^ TAMs secrete IL‐6. Analysis of published ChIP‐seq data demonstrated that treatment with the AR agonist R1881 enhanced AR binding to the IL‐6 promoter in THP‐1 cells (Figure S5A). This led us to examine whether AR, a key transcription factor in PCa, directly regulates IL‐6 at the transcriptional level. ChIP‐qPCR confirmed AR binding to the IL‐6 promoter and direct transcriptional activation in both human (THP‐1) and mouse (Raw264.7) macrophage‐like cell lines (Figure 5A,B). Using the JASPAR database, we identified three potential AR binding sites within the IL‐6 promoter (Figure 5C). Mutational analysis combined with dual‐luciferase reporter assays revealed that mutation 1 significantly impaired AR binding, indicating that AR regulates IL‐6 transcription primarily through recognition and binding to the sequence TGGCACAGAGAGCAA in human PBMCs and THP‐1 cells (Figure 5D,E). Furthermore, analysis of single‐cell datasets (GSE206962 and GSE137829) showed significant co‐localization of AR and IL‐6 in TAMs (Figure 5I,J). This finding was validated by mIF in human CRPC samples (Figure 5F) and in two ENZ‐resistant mouse models (Figure 5G,H), where IL‐6 was prominently localized around AR^+^ TAMs, supporting the notion of paracrine IL‐6 secretion by AR^+^ TAMs. Collectively, these findings establish that AR directly binds to the IL‐6 promoter to drive its transcriptional activation in TAMs, revealing a cell‐intrinsic regulatory mechanism that underlies the co‐expression of AR and IL‐6 within the prostate TME.

### 2.6 AR^+^ TAMs Drive Therapy Resistance via IL‐6‐Mediated Activation of the JAK2/STAT3 Pathway

**FIGURE 6:**
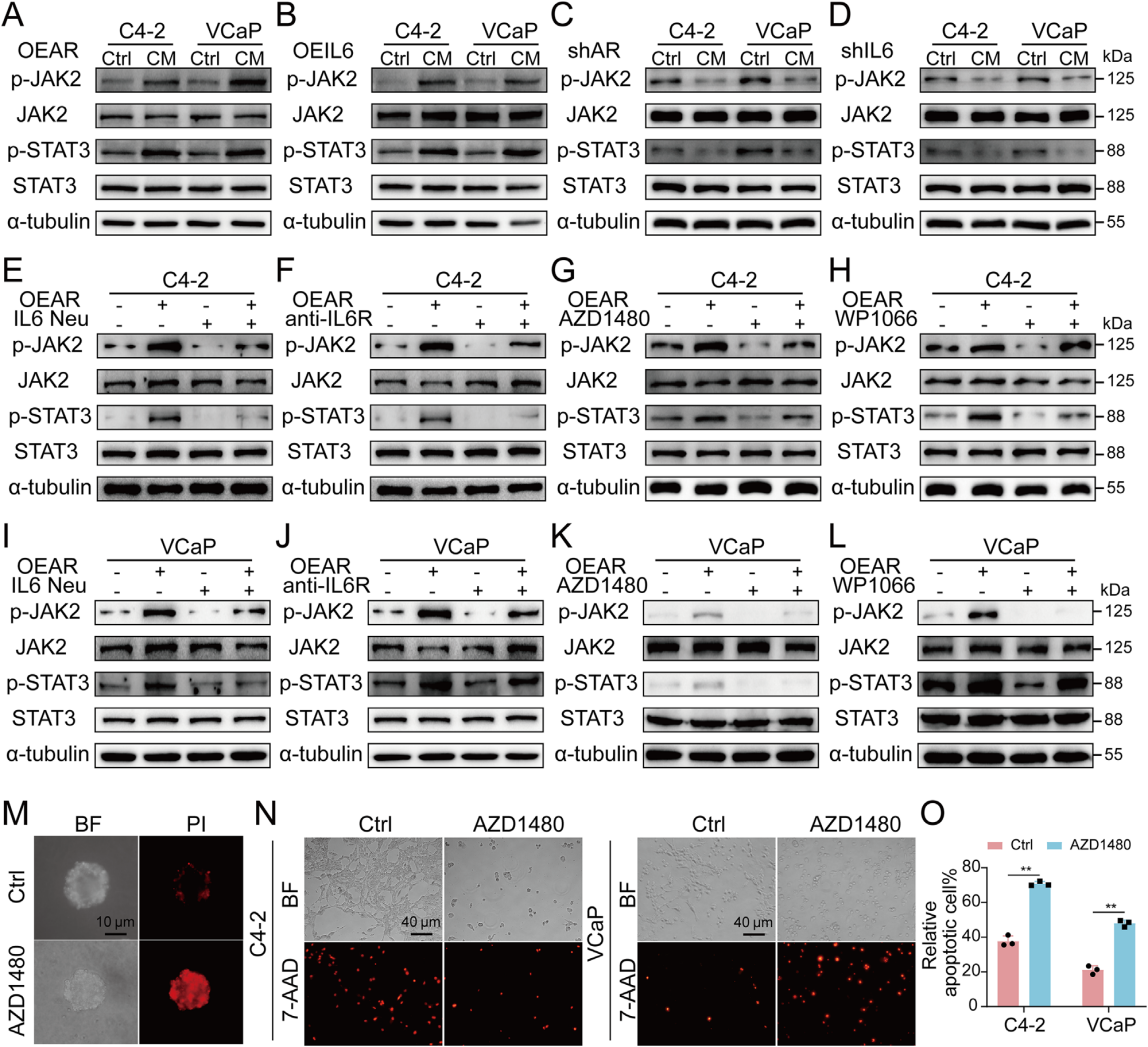
AR^+^ TAMs drive therapy resistance via IL‐6‐mediated activation of the JAK2/STAT3 pathway. (A–D) WB analysis evaluating the protein levels of p‐JAK2, JAK2, p‐ STAT3, and STAT3 in C4‐2 and VCaP cells. Tumor cells were treated with CM derived from AR‐overexpressing (OEAR, A), IL‐6‐overexpressing (OEIL‐6, B) TAMs, or TAMs with AR knockdown (shAR, C) or IL‐6 knockdown (shIL‐6, D). (E–L) WB analysis of the aforementioned JAK2/STAT3 pathway components in C4‐2 (E–H) and VCaP (I–L) cells. Cells were exposed to CM from OEAR TAMs, with or without IL‐6 neutralizing antibody (IL‐6 Neu, E, I), anti‐IL‐6 receptor antibody (anti‐IL‐6R, F, J), JAK2 inhibitor (AZD1480, G, K), or STAT3 inhibitor (WP1066, H, L). (M–O) Analysis of apoptosis in organoids (M), C4‐2, and VCaP cells (N) treated with ENZ and CM from OEAR TAMs, with or without AZD1480. (O) Quantification of the relative apoptotic cell percentage from (N). Scale bar = 10 µm (M), Scale bar = 40 µm (N).

To explore how secreted IL‐6 contributes to therapy resistance, we performed enrichment analysis and observed significant activation of the JAK/STAT pathway following IL‐6 stimulation (Figure S5B). Treatment of tumor cells with CM from AR^+^ TAMs resulted in marked activation of the JAK2/STAT3 pathway (Figure 6A). Similarly, supernatant from IL‐6‐overexpressing TAMs enhanced JAK2/STAT3 signaling (Figure 6B), whereas knockdown of AR or IL‐6 reduced phosphorylation levels (Figure 6C,D). Moreover, induction of pJAK2 and pSTAT3 by AR^+^ TAMs‐CM was suppressed by IL‐6 neutralizing antibody, IL‐6 receptor blockade, or JAK2/STAT3 pathway inhibitors (AZD1480 and WP1066) (Figure 6E–L). Under ENZ treatment, the induction of pJAK2 and pSTAT3 by AR^+^ TAMs‐CM was counteracted by the JAK2 inhibitor AZD1480, thereby successfully restoring cellular sensitivity to apoptosis (Figure 6M–O). Together, these findings demonstrate that secreted IL‐6 from AR^+^ TAMs critically mediates therapy resistance by driving persistent JAK2/STAT3 signaling, which sustains pro‐survival signaling and apoptosis evasion under ENZ treatment.

### 2.7 Targeting IL‐6 Overcomes ENZ Resistance and Suppresses Tumor Growth in CRPC

**FIGURE 7:**
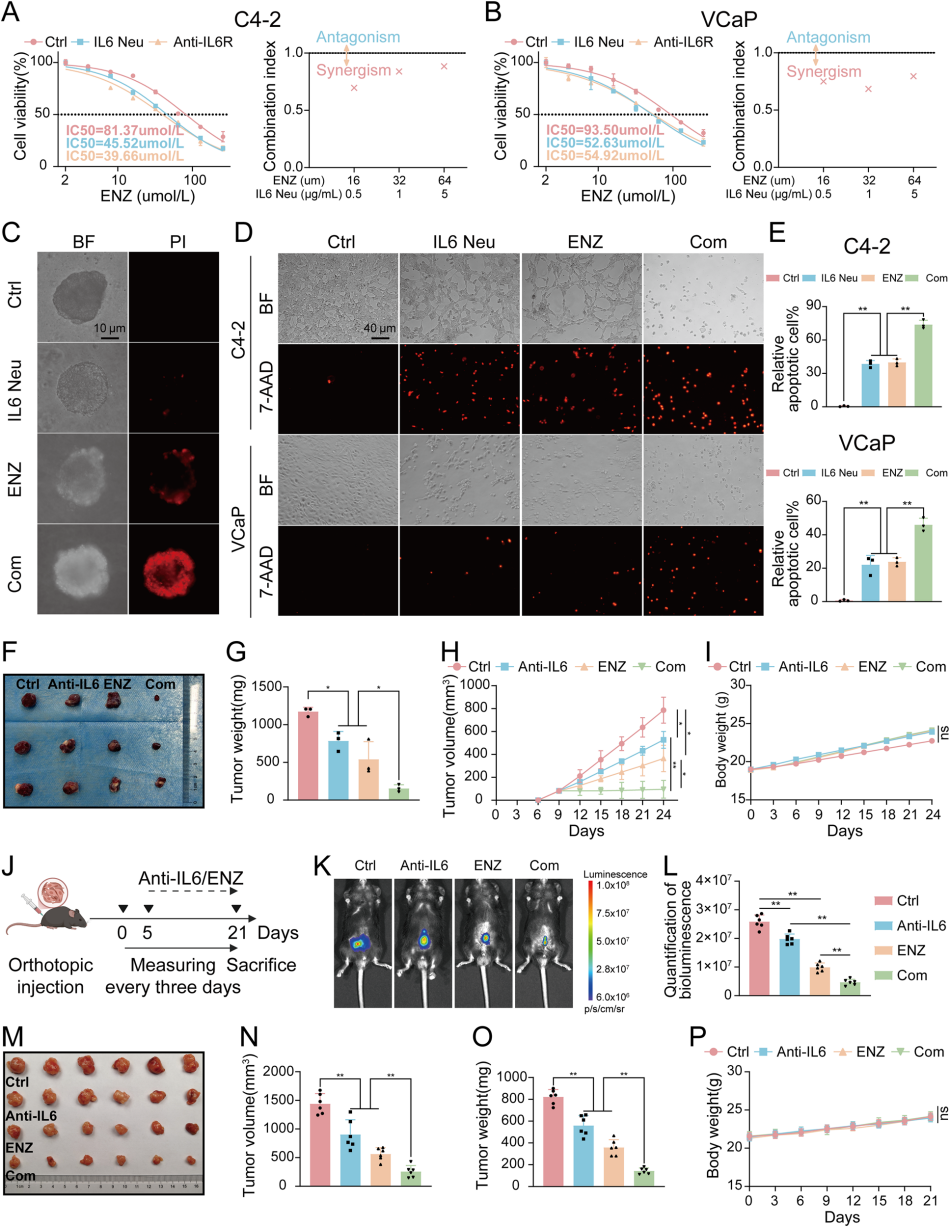
Targeting IL‐6 overcomes ENZ resistance and suppresses tumor growth in CRPC. (A, B) Dose‐response curves evaluating the cell viability of C4‐2 (A) and VCaP (B) cells treated with ENZ in the presence of an IL‐6 neutralizing antibody (IL‐6 Neu) or an anti‐IL‐6 receptor antibody (Anti‐IL‐6R). Right panels: Combination index (CI) plots demonstrating the synergism (CI < 1) between ENZ and IL‐6 Neu. (C) Representative bright‐field (BF) and PI staining of *Pten* ^PC‐/−^; *Trp53* ^PC‐/−^ organoids co‐cultured with AR^+^ TAMs and treated with vehicle control (Ctrl), IL‐6 Neu, ENZ, or their combination (Com). Scale bar = 10um. (D, E) Representative 7‐AAD staining images (D) and quantification (E) of apoptotic C4‐2 and VCaP cells stimulated by AR^+^ TAMs after treatment with Ctrl, IL‐6 Neu, ENZ, or Com. Scale bar = 40um. (F–I) Therapeutic efficacy in CRPC PDX models, showing representative macroscopic images of excised tumors (F), terminal tumor weights (G), tumor growth curves (H), and systemic body weights over time (I) following treatment with ctrl, anti‐IL‐6 antibody, ENZ, or Com. (J–P) Validation in the orthotopic murine model. (J) Schematic illustration of the experimental design. (K, L) Representative bioluminescence imaging (K) and quantification (L) of tumor burden. (M–P) Corresponding gross images of excised tumors (M), terminal tumor volumes (N) and weights (O), and murine body weights (P).

Given that IL‐6 derived from AR^+^ TAMs activates the JAK2/STAT3 pathway to suppress apoptosis and confer resistance to ENZ, we next asked whether disrupting IL‐6 signaling could restore ENZ sensitivity in cancer cells. In vitro, stimulation with CM from AR^+^ TAMs was counteracted by IL‐6 neutralization with an antibody or blockade of the IL‐6 receptor, which significantly enhanced ENZ responsiveness (Figure 7A,B). Co‐treatment with an IL‐6 neutralizing antibody and ENZ resulted in CI values below 1, indicating synergistic effects (Figure 7A,B). Moreover, in organoids derived from *Pten/p53* double‐knockout organoids under AR^+^ TAMs stimulation, the combination of IL‐6 neutralizing antibody and ENZ markedly increased tumor cell death (Figure 7C). Apoptosis analysis by 7‐AAD staining and flow cytometry further confirmed a significant increase in apoptotic cells following combination treatment (Figure 7D,E; Figure S6A,B).

More importantly, in patient‐derived xenograft (PDX) models generated from clinical CRPC samples and engrafted into NCG mice, the combination of anti‐IL‐6 and ENZ led to a pronounced reduction in both tumor weights and volumes (Figure 7F–H), without significant changes in body weights observed across the four experimental groups (Figure 7I). Similarly, in an orthotopic model established in wild‐type mice (Figure 7J), bioluminescence imaging (Figure 7K,L), gross macroscopic examination (Figure 7M), and measurements of tumor volumes and weights (Figure 7N,O) all revealed substantial tumor regression in the combination treatment group, without notable alterations in body weights (Figure 7P). Histological assessment of sections from both in vivo models demonstrated that combined therapy strongly suppressed Ki‐67 expression and increased cleaved caspase‐3 levels (Figure S6C–F). Furthermore, to obtain direct biochemical evidence of target engagement in vivo, we assessed systemic IL‐6 levels and intra‐tumoral STAT3 activation. ELISA analysis revealed that while anti‐IL‐6 or ENZ monotherapies reduced serum IL‐6 to varying degrees, the combination treatment achieved the most significant suppression (Figure S6G). Consistent with these systemic alterations, WB analysis of harvested tumor tissues confirmed that the combination therapy elicited the most profound blockade of STAT3 phosphorylation (Figure S6H). Collectively, these results underscore that anti‐IL‐6 therapy in combination with ENZ represents a promising therapeutic strategy for CRPC.

## 3 Discussion

The emergence of therapeutic resistance in CRPC remains a critical clinical challenge [32, 33]. While SGAT drugs such as ENZ initially elicit robust responses, acquired resistance is virtually inevitable, highlighting the limitations of exclusively targeting cancer cell‐intrinsic pathways [34, 35]. In this study, we identify an extrinsic resistance mechanism driven by a distinct subpopulation of AR^+^ TAMs. Rather than expressing AR endogenously, these TAMs acquire the functional protein via ANXA2‐dependent phagocytosis of prostate tumor cells. This internalized AR subsequently acts as a transcriptional driver for IL‐6, establishing a paracrine loop that shields cancer cells from ENZ‐induced apoptosis through the JAK2/STAT3 axis.

A central finding of this work is the non‐canonical acquisition of AR by TAMs. Given the negligible endogenous AR expression in myeloid cells, we explored alternative pathways and identified phagocytosis as the primary driver. Comparative profiling between phagocytic and non‐phagocytic macrophages revealed that AR protein enrichment is strictly confined to the phagocytic subset and heavily dependent on ANXA2, a critical regulator of membrane‐cytoskeleton dynamics [36]. Knockdown of ANXA2 significantly impaired both phagocytic activity and the emergence of AR^+^ TAMs, thereby establishing a molecular link between phagocytic machinery and the formation of this functionally distinct macrophage population. While our data establish ANXA2 as a principal mediator of this engulfment, macrophage phagocytosis is a highly coordinated process involving a broader network of surface receptors. Indeed, our membrane proteomic profiling identified other phagocytosis‐associated candidates, such as SND1, among the top‐enriched proteins. However, SND1 has been reported to inhibit phagocytosis by reinforcing the anti‐phagocytic CD47‐SIRPα axis [37]. Given our objective to investigate the mechanisms actively driving tumor cell internalization and the subsequent generation of AR^+^ TAMs, we prioritized the positive regulator ANXA2. Nevertheless, the potential cooperative roles of other canonical scavenger receptors in mediating tumor cell phagocytosis within the TME warrant further investigation. These observations suggest a model in which the engulfment of tumor cells by TAMs is not merely an internalization process, but a pivotal event that drives TAMs subset specification.

For this extrinsically acquired AR to exert functional dominance, it must evade complete phagolysosomal degradation and translocate into the macrophage nucleus [38]. In professional phagocytes, internalized macromolecules can bypass lysosomal destruction via phagosome‐to‐cytosol escape, a process classically required for antigen cross‐presentation [39, 40]. Recent evidence demonstrates that professional phagocytes actively permeabilize their own endocytic compartments using endogenous pore‐forming effectors, such as Perforin‐2 or the Sec61 translocon, facilitating the release of luminal contents into the cytosol [41, 42]. We postulate that the exogenously phagocytosed AR exploits these host‐driven endosomal escape pathways to access the macrophage cytosol. Subsequently, the intrinsic nuclear localization signal of the AR protein engages the macrophage's importin transport machinery, ensuring its successful nuclear import to transactivate the IL‐6 promoter.

TAMs promote tumor progression and confer therapy resistance through diverse secretory functions [18, 43]. In this study, we identify AR^+^ TAMs as a major source of IL‐6 in the CRPC microenvironment. Previous studies have implicated IL‐6 in PCa pathogenesis and resistance to ENZ, with evidence suggesting that it engages in bidirectional crosstalk with AR signaling to promote a stem‐like, treatment‐resistant phenotype [44, 45]. Nevertheless, the cellular sources and regulatory mechanisms controlling IL‐6 production have remained poorly understood. We demonstrate that the acquisition of AR enables macrophages to function as a principal source of IL‐6. This mechanism diverges from classical cytokine production, as IL‐6 expression is directed by a transcription factor acquired extrinsically through phagocytosis rather than through endogenous synthesis. Our findings broaden the understanding of cytokine regulation in the TME by revealing how macrophages co‐opt transcription factors from cancer cells to reprogram their secretory output and drive therapy resistance.

Despite the preclinical efficacy of co‐targeting the IL‐6/JAK2/STAT3 axis, the clinical translation of IL‐6 blockade in CRPC entails potential challenges. Systemic inhibition of IL‐6 signaling, using agents such as siltuximab or tocilizumab, has been associated with adverse events, including immunosuppression, hepatotoxicity, and an increased risk of severe infections, owing to the pleiotropic roles of IL‐6 in normal physiological homeostasis and immune responses [46]. Consequently, adopting a precision medicine approach is imperative to maximize the therapeutic index. Given the profound heterogeneity of the TME, patient stratification based on the intra‐tumoral density of AR^+^ TAMs could serve as a robust predictive biomarker. Selecting patients with an AR^+^ TAMs‐enriched TME ensures that the combination of ENZ and IL‐6 blockade is administered specifically to those who rely on this paracrine resistance mechanism, thereby mitigating unnecessary systemic toxicities in non‐responders and precisely overcoming TAMs‐mediated therapy resistance.

In conclusion, this study reveals a non‐autonomous resistance circuit in CRPC driven by phagocytosis‐mediated transcription factor hijacking. By linking ANXA2‐dependent tumor clearance to the generation of AR^+^ TAMs and subsequent IL‐6 paracrine signaling, our findings provide a compelling mechanistic basis for dual AR and IL‐6 pathway inhibition in targeted patient populations.

## 4 Materials and Methods

### 4.1 Clinical Samples and Mouse Models

Formalin‐fixed, paraffin‐embedded (FFPE) tissue specimens from 153 PCa patients, including 18 cases resistant to SGAT, were obtained with informed consent. The study protocol was approved by the Institutional Review Board of Sun Yat‐sen Memorial Hospital, Sun Yat‐sen University (No. SYSKY‐2023‐925‐01). Comprehensive clinical data, including age, tumor grade, TNM stage, and PSA levels, were collected for the cohort and summarized in Table S1. To establish an ENZ‐resistant CRPC model, *Pten/Trp53* double‐knockout mice with spontaneous PCa underwent surgical castration to induce androgen deprivation, followed by ENZ administration (10 mg/kg, i.p., 3 times/week, S1250, Selleck, Shanghai, China) [47]. For the organoid‐based orthotopic model, primary prostate tumor tissues were isolated from *Pten/Trp53*‐deficient mice and cultured as organoids. These organoids were resuspended in 20 µL Matrigel (356231, Corning, USA) and orthotopically injected into the anterior prostate lobe of syngeneic 8‐week‐old wild‐type C57BL/6J male mice (BesTest, Zhuhai, China). Once the mice bearing orthotopic tumors reached 10 weeks of age, they underwent simultaneous surgical castration and ENZ treatment to induce the resistant phenotype. Under continuous ENZ selection pressure, tumors demonstrating progressive growth were classified as ENZ‐resistant, while those exhibiting significant volume regression or growth arrest were defined as ENZ‐sensitive. To establish orthotopic models, RM1‐luciferase cells were mixed with either AR‐positive luciferase‐expressing Raw264.7 cells or AR‐positive IL‐6‐knockdown (KD) luciferase‐expressing Raw264.7 cells at a 4:1 ratio in PBS and Matrigel, followed by injection into the anterior prostate of C57BL/6 mice. For PDX models, tumor fragments from CRPC patients were subcutaneously implanted into NCG mice (GemPharmatech, Jiangsu, China). Mice were randomized into treatment groups receiving ENZ (10 mg/kg, i.p., 3 times/week), anti‐IL‐6 antibody (10 mg/kg, i.p., once every two days, Selleck, Shanghai, China), or their combination. Tumor progression was monitored via bioluminescence imaging and caliper measurements. All animal experiments were conducted in accordance with the protocol approved by the Institutional Animal Care and Use Committee of Sun Yat‐Sen University (approval number AP20230225).

### 4.2 Cell Culture and Transfection

The human CRPC cell lines C4‐2 and VCaP, the murine PCa cell line RM1, human monocytic THP‐1 cells, murine RAW264.7 macrophages, and human embryonic kidney HEK293T cells were acquired from the American Type Culture Collection. Human peripheral blood mononuclear cells (PBMCs) were isolated from patients diagnosed with PCa. All cell lines were maintained at 37°C under a humidified atmosphere of 5% CO_2_, cultured in either RPMI‐1640 or DMEM medium (Gibco, USA) supplemented with 10% fetal bovine serum and 1% penicillin‐streptomycin (Gibco, USA). To establish knockdown (KD) or overexpression (OE) of AR, ANXA2, or IL‐6, specific shRNAs and expression plasmids (IGE Biotechnology, China) were constructed and verified by sequencing (Table S2). Transfections were performed using the X‐tremeGENE HP DNA Transfection reagent (6366244001, Roche, Switzerland) according to the manufacturer's instructions.

### 4.3 Phagocytosis Assays and Cell Sorting

To evaluate phagocytosis, GFP‐expressing tumor cells were co‐cultured with RAW264.7 macrophages at a 2:1 ratio for 48 h [48]. Following incubation, cells were harvested and sequentially stained with Ghost dye (Tonbo, China), CD45, CD11b, and F4/80 antibodies (Elabscience, China). Phagocytosis was quantified by detecting GFP signal within macrophages using both flow cytometry and confocal microscopy. Macrophages that had engulfed tumor cells, identified as GFP^+^ F4/80^+^, were classified as TCPMs, while those that had not, defined as GFP^−^ F4/80^+^, were designated as TCNPMs. These populations were isolated using fluorescence‐activated cell sorting for further analysis.

### 4.4 Cell Viability and Functional Assays

Cell viability was assessed using the CCK‐8 assay (ApexBio, USA). C4‐2 and VCaP cells were seeded in 96‐well plates and treated with CM collected from 48h cultures of AR^+^ TAMs, which was concentrated tenfold using Beckman Optima XE‐100 Ultracentrifuge. In drug sensitivity experiments, cells were exposed to a range of concentrations of ENZ, rhIL‐6 (RP00201, Abclonal, Wuhan, China), IL‐6‐neutralizing antibody (501125, 504501, Biolegend, USA), or an IL‐6 receptor blocker (S412003, Aladdin, Shanghai, China). Following treatment, 10 µL of CCK‐8 reagent was added to each well, and absorbance was measured at 450 nm. Drug interactions were evaluated with CalcuSyn software, whereby a combination index value less than 1 was interpreted as synergistic, equal to 1 as additive, and greater than 1 as antagonistic. For colony formation assays, 1000 cells per well were plated in 6‐well plates and subjected to the indicated treatments for two weeks. The resulting colonies were fixed with 4% paraformaldehyde, stained with 0.1% crystal violet, and quantified using image analysis software. Apoptosis was evaluated via flow cytometry with Annexin V‐FITC and PI co‐staining (E‐CK‐A211, Elabscience, Wuhan, China) [49]. In parallel, fluorescent microscopy using 7‐AAD staining (KeyGEN, Jiangsu, China) was performed to visualize and quantify apoptotic cells. Organoid viability was determined by PI assay.

### 4.5 qRT‐PCR and ELISA Analysis

Total RNA was extracted from cultured cells using TRIzol reagent (G3013, Servicebio, Wuhan, China) and quantified using a NanoDrop spectrophotometer. First‐strand cDNA was synthesized from 1 µg of total RNA using a reverse transcription kit (R223‐01, Vazyme, China). Quantitative real‐time PCR (qRT‐PCR) was performed with SYBR Green Premix Ex Taq II on a QuantStudio DX Real‐Time PCR System (500‐102, GOONIE, China). All reactions were conducted in triplicate, and gene expression levels were normalized to GAPDH and calculated via the 2^−ΔΔCt^ method. Primer sequences are listed in Table S2. Secreted IL‐6 levels in cell culture supernatants and conditioned media, along with murine IL‐6 levels in peripheral blood serum from animal models, were quantified using species‐specific ELISA kits (ml058097 ‐ 1, mlbio, Shanghai, China, for human; E‐EL‐M0044, Elabscience, Wuhan, China, for mouse) strictly following the manufacturers’ instructions. Absorbance was measured at 450 nm, and cytokine concentrations were determined based on standard curves.

### 4.6 Western Blot and Mass Spectrometry Analysis

Protein lysates were extracted using RIPA buffer (P0013B, Beyotime, China) supplemented with a protease inhibitor cocktail (HY‐K0010, MCE, USA) and phosphatase inhibitors (G2007, Servicebio, China). For immunoblotting [50], membranes were incubated with specific primary antibodies targeting AR (YM8602, Immunoway, USA), ANXA2 (11256‐1‐AP, Proteintech, Wuhan, China), IL‐6 (21865‐1‐AP, Proteintech, China), as well as key lysosomal and phagocytosis‐associated proteins, including CLTC (66487‐1‐Ig, Proteintech, China), LIPA (12956 ‐ 1 ‐ AP, Proteintech, China), and LAMP2 (66301 ‐ 1 ‐ Ig, Proteintech, China) and LAPTM5 (A17995, Abclonal, China). Additionally, components of the JAK2/STAT3 signaling pathway were examined (A11497, AP0531, A22434, Abclonal; F1212, selleck, China). For membrane proteomic profiling, sorted macrophages were lysed and fractionated using the Minute Plasma Membrane/Protein Isolation and Cell Fractionation Kit (SM‐005, Invent, USA). Enriched membrane proteins were then subjected to liquid chromatography tandem mass spectrometry analysis (FITGENE Biotechnology, China).

### 4.7 Immunofluorescence and Immunohistochemistry

FFPE sections were subjected to mIF staining using a 7‐color TSA mIHC Kit (10300100100, Panovue, China). The primary antibodies employed were as follows: AR, CD68 (97778, CST, USA), F4/80 (70076, CST, USA), and IL‐6. Immunofluorescence labeling for cellular markers was performed using antibodies against F4/80 and IL‐6R (66855‐1‐Ig, Proteintech, China). IHC was conducted according to the instructions of the UltraSensitive SP detection kit (KIT‐9720, Maxim, Fuzhou, China), incorporating antibodies against Ki‐67 (27309‐1‐AP, 28074‐1‐AP, Proteintech, China), caspase‐3 (GB11767C, Servicebio, China), and cleaved caspase‐3 (GB11532, Servicebio, China) [49]. All images were acquired on a Leica SP8 STED 3X confocal microscope and quantitatively analyzed using ImageJ software.

### 4.8 Chromatin Immunoprecipitation and Luciferase Assays

Chromatin immunoprecipitation (ChIP) assays were conducted with an antibody against AR using an enzymatic ChIP kit (P2078, Beyotime, Shanghai, China) [51]. The immunoprecipitated DNA was subjected to quantitative PCR analysis with primers specific to the IL‐6 promoter region. Putative AR binding motifs within this region were predicted via the JASPAR database. To functionally validate these sites, segments of the IL‐6 promoter were cloned into a luciferase reporter vector. In addition, site‐directed mutagenesis was employed to generate mutations (IGE Biotechnology, China) in the predicted AR binding sites for subsequent luciferase assays (11402ES60, Yeasen, China).

### 4.9 Bioinformatics Analysis

scRNA‐seq datasets were obtained from the Gene Expression Omnibus (GEO) under accession codes GSE206962 and GSE137829. Data processing, including quality control, normalization, and clustering, was performed using the R package Seurat. Cells expressing fewer than 200 genes or exhibiting mitochondrial gene content exceeding 20% were excluded from the analysis. The Harmony algorithm was applied to mitigate batch effects. Correlation between ANXA2 expression and markers associated with macrophages and phagocytosis was evaluated using the GEPIA online tool (http://gepia.cancer‐pku.cn/). Publicly available ChIP‐seq data from THP‐1 cells treated with either DMSO or the synthetic androgen R1881 were retrieved from GEO (GSE131381) [10]. AR binding peaks from these data were visualized and analyzed using the Integrative Genomics Viewer. Furthermore, bulk RNA sequencing was performed on AR‐overexpressing THP‐1 cells and rhIL‐6‐stimulated PCa cells, with DEGs identified via the DESeq2 package.

### 4.10 Statistical Analysis

Data are presented as the mean ± SEM from a minimum of three independent experiments. All statistical analyses were performed using GraphPad Prism software (version 10.0). Differences between the two groups were assessed using the two‐tailed Student's t‐test, whereas one‐way or two‐way ANOVA was utilized for multiple comparisons. Survival analysis was conducted using the Kaplan–Meier method with log‐rank testing. Univariate and multivariate Cox proportional hazards models were applied to identify independent prognostic factors. Statistical significance was defined as *p* < 0.05.

## Author Contributions

Y.L., H.H., B.C., K.X., and S.P. designed this study. Y.L., T.L., L.L., and Q.S. contributed to the experimental design. Y.L., T.L., D.L., S.H., Z.L., W.L., Y.O., and T.D. performed the experiments and performed the data analysis. Y.L., T.L., and B.C. wrote the manuscript. H.H., B.C., K.X., and S.P. provided funding for the research, with all the authors contributing to providing feedback.

## Conflicts of Interest

The authors declare no conflicts of interest.

## Supporting information




**Supporting File**: advs75290‐sup‐0001‐SuppMat.docx.

## Data Availability

The data that support the findings of this study are available from the corresponding author upon reasonable request.
