# Supplementary material for: ANXA2‐mediated Phagocytosis Generates AR+ Macrophages to Confer Enzalutamide Resistance in Prostate Cancer
Source: Adv Sci (Weinh). 2026 Apr 16;13(38):e75290. doi: 10.1002/advs.75290 (PMC13335082; doi:10.1002/advs.75290)
Supplement: Supplementary file 1 — Supporting File: advs75290‐sup‐0001‐SuppMat.docx. [file ADVS-13-e75290-s001.docx]

**
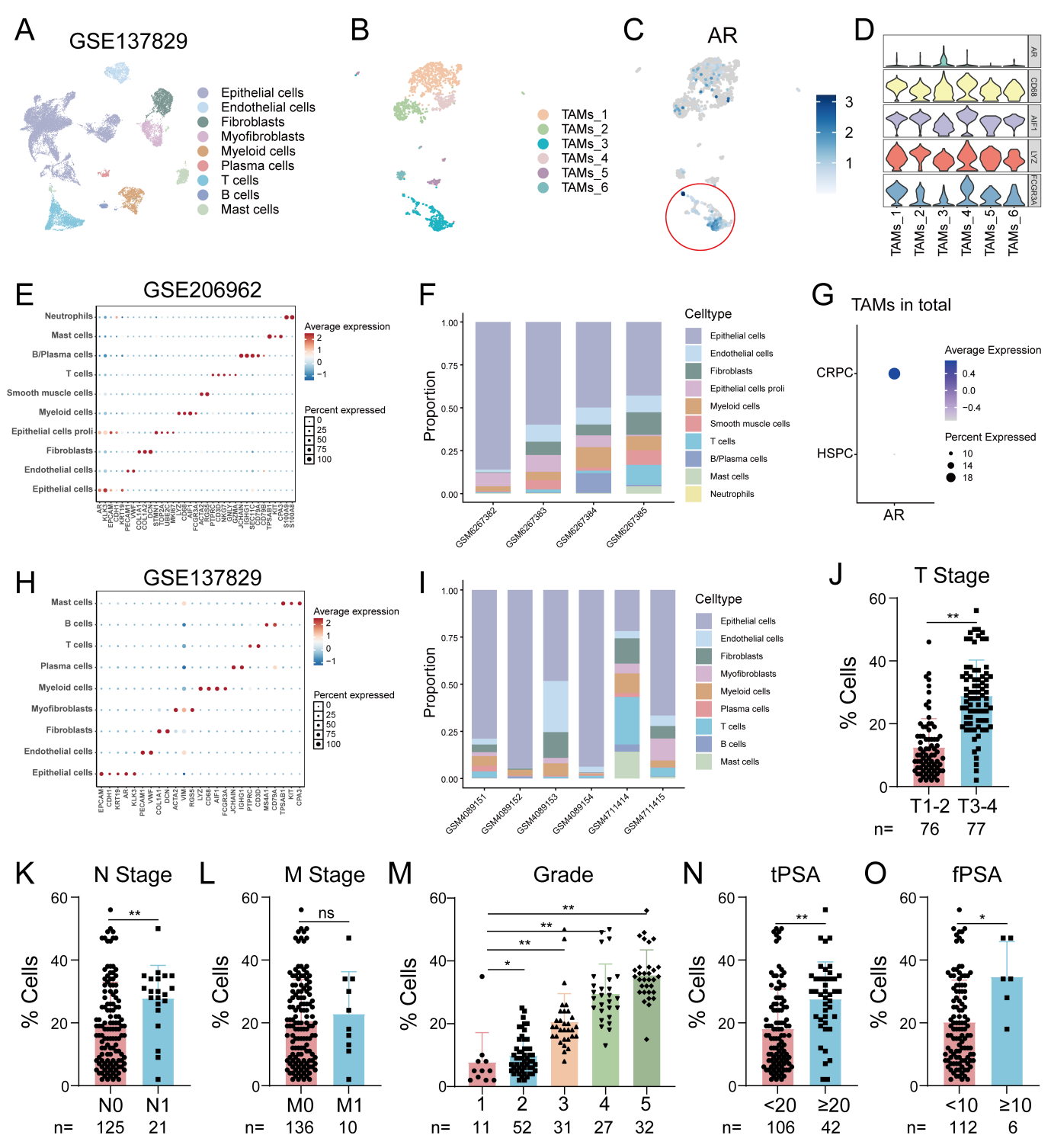
**

**Figure S1**. Enrichment of AR^+^ TAMs in the CRPC microenvironment correlates with adverse clinical-pathological features.

(A) UMAP projection of major cell types identified in the GSE137829 dataset derived from CRPC patients. (B) UMAP plot highlighting the distinct TAMs subpopulations (TAMs_1–TAMs_6). (C) UMAP visualization of AR expression distribution within the TAMs subpopulations. (D) Violin plots displaying the expression levels of AR and canonical myeloid/macrophage markers across the six TAMs subpopulations. (E) Dot plot illustrating the expression profiles of lineage-specific marker genes used for cell type annotation in the GSE206962 dataset. (F) Stacked bar plot showing the proportion of different cell types. (G) Dot plot comparing AR expression levels and the percentage of AR-expressing TAMs between CRPC and hormone-sensitive prostate cancer (HSPC) samples. (H) Dot plot showing marker gene expression profiles for distinct cell populations in the GSE137829 dataset. (I) Stacked bar plots displaying the relative cellular composition across individual samples. (J–O) Scatter bar plots evaluating the correlation between the proportion of AR^+^ TAMs and clinical-pathological parameters in PCa patients, including T stage (T1-2 vs. T3-4) (J), N stage (N0 vs. N1) (K), M stage (M0 vs. M1) (L), tumor grade (1–5) (M), tPSA (<20 vs. ≥20ng/mL) (N), and fPSA (<10 vs. ≥10ng/mL) (O).


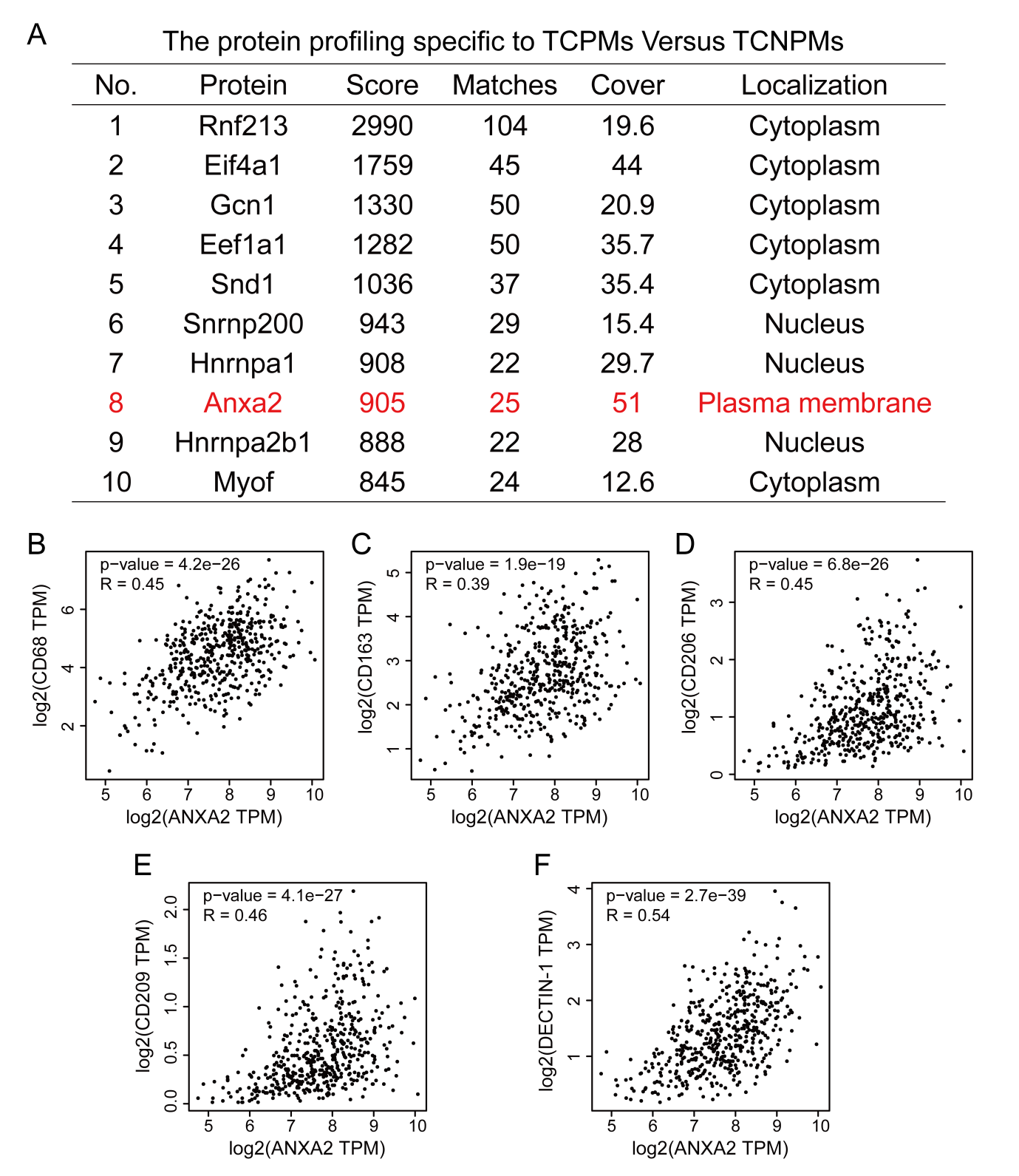


**Figure S2**. Proteomic identification of ANXA2 and its correlation with macrophage markers.

(A) List of top 10 identified proteins with their score, number of peptide matches, sequence coverage, and subcellular localization. ANXA2 is highlighted in red. (B–F) Scatter plots illustrating the positive correlation between ANXA2 mRNA expression and canonical macrophage markers, including CD68 (B), CD163 (C), CD206 (D), CD209 (E), and DECTIN-1 (F) based on the GEPIA database.


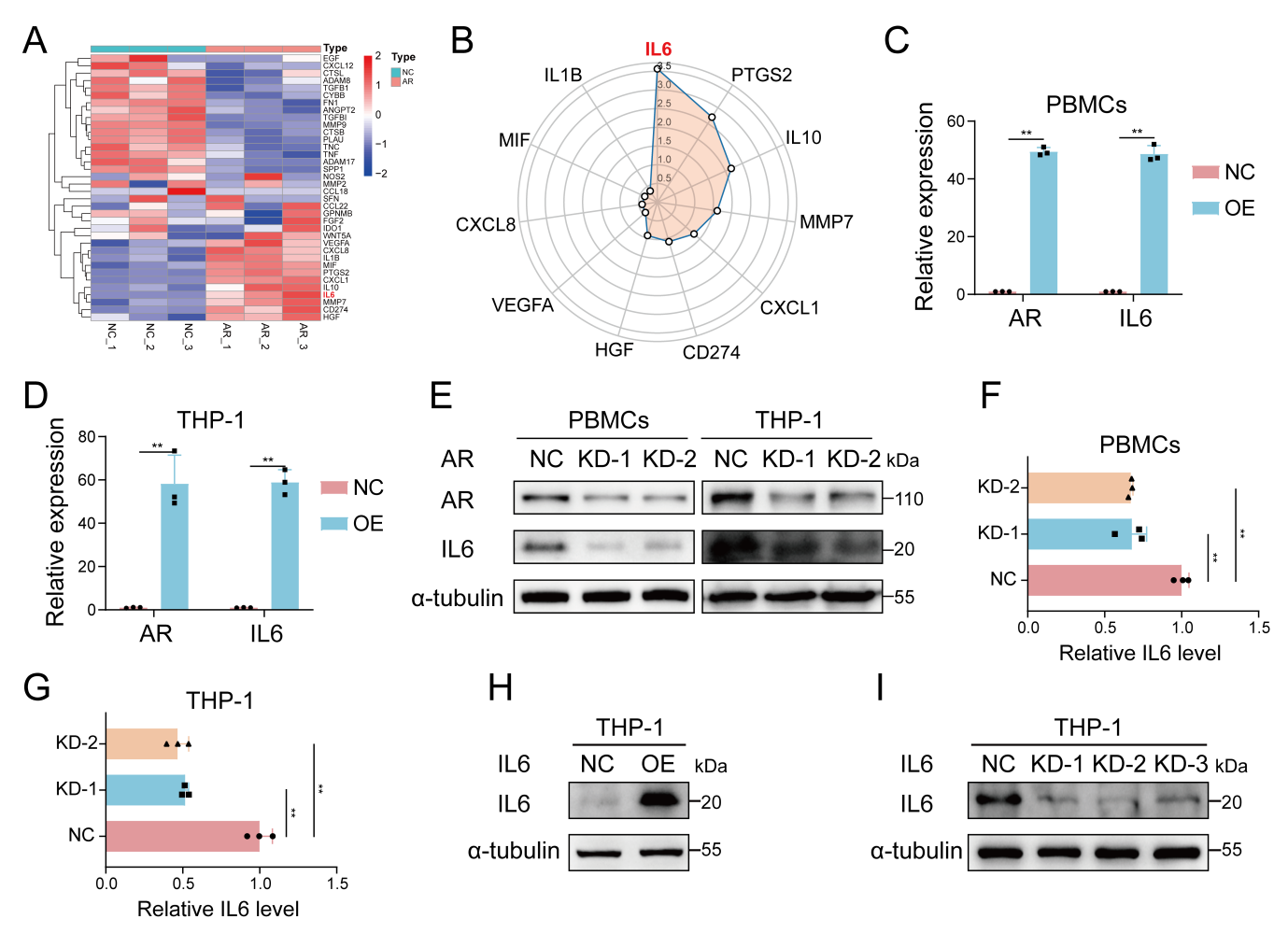


**Figure S3**. AR regulates IL-6 expression and secretion in TAMs.

(A) Heatmap depicting the differential expression profiles of TAMs-associated secretory factors in THP-1 cells following AR overexpression (AR) compared to the negative control (NC). (B) Radar chart illustrating the expression fold changes of selected macrophage-derived cytokines, highlighting IL-6 as the most prominently upregulated gene upon AR OE. (C, D) Relative mRNA expression of AR and IL-6 in PBMCs (C) and THP-1 cells (D) with AR OE or NC. (E) Western blot analysis of AR and IL-6 protein levels in PBMCs and THP-1 cells following AR knockdown (KD). (F, G) Quantification of secreted IL-6 levels in the culture supernatants of PBMCs (F) and THP-1 cells (G) subjected to AR KD. (H, I) Western blot validation of IL-6 protein levels in THP-1 cells upon IL-6 OE (H) and IL-6 KD (I).


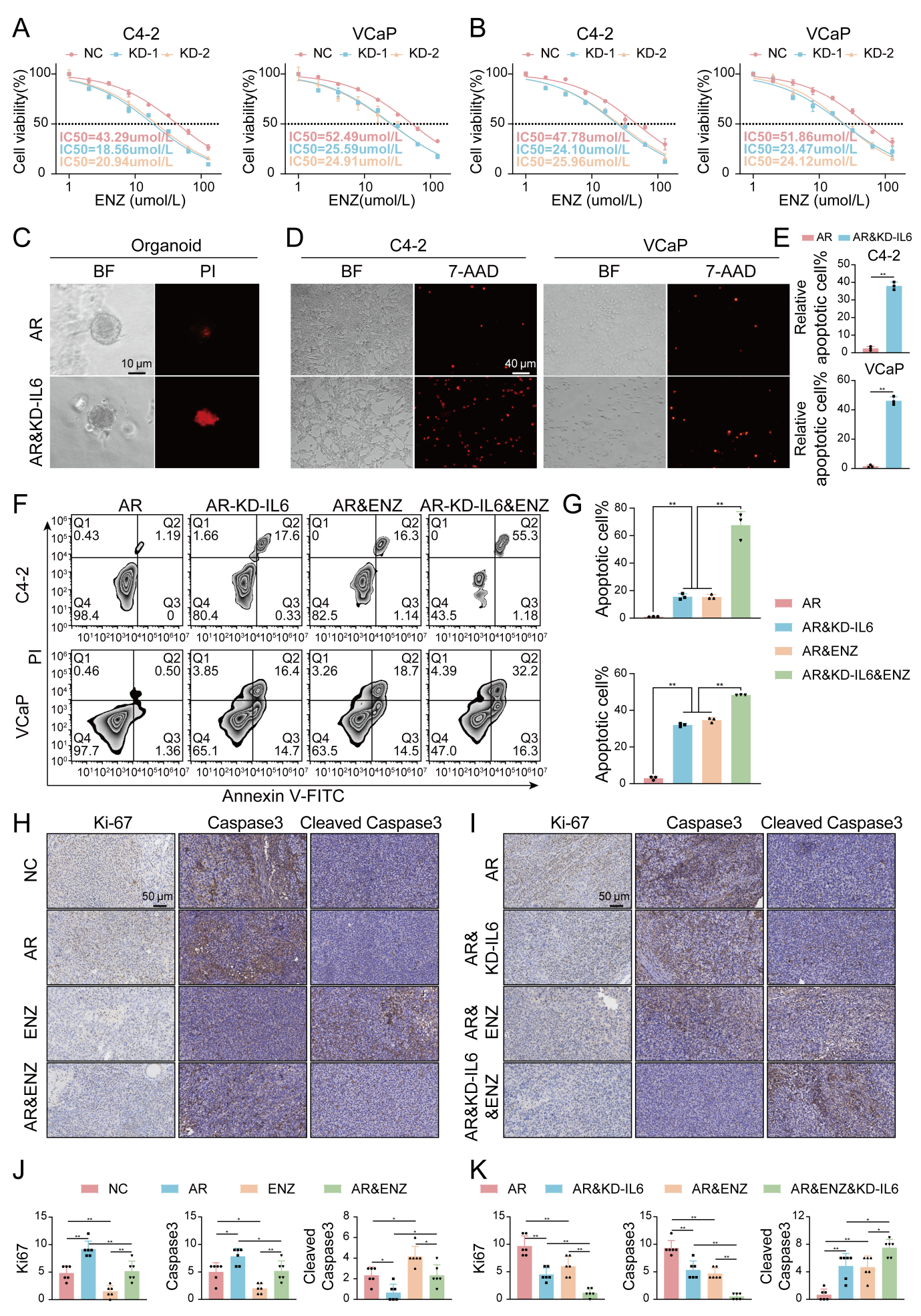


**Figure S4**. IL-6 is crucial for AR^+^ TAMs-mediated ENZ resistance and anti-apoptotic effects.

(A, B) Dose-response curves showing the viability of C4-2 and VCaP cells treated with varying concentrations of ENZ, cultured in conditioned media (CM) derived from AR-knockdown (KD-1, KD-2) TAMs (A) or IL6-KD TAMs (B), compared to control TAMs (NC). (C) PI staining of *Pten/p53* double-knockout prostate organoids treated with ENZ and CM from AR^+^ TAMs with IL-6 knockdown (AR&KD-IL-6) or control AR^+^ TAMs (AR). Scale bar = 10um. (D, E) 7-AAD fluorescence staining (D) and corresponding quantification (E) of apoptosis in C4-2 and VCaP cells co-cultured with CM from AR&KD-IL6 or AR groups. Scale bar = 40um. (F, G) Flow cytometric analysis of apoptosis (F) and quantitative comparison (G) in C4-2 and VCaP cells across different treatment groups: AR, AR&KD-IL6, AR&ENZ, and AR&KD-IL6&ENZ. (H-K) Representative IHC images (H, I) and corresponding quantitative analysis (J, K) of Ki-67, Caspase-3, and Cleaved Caspase-3 in tumor xenografts from Figure 4E and 4L. Scale bar = 50um.


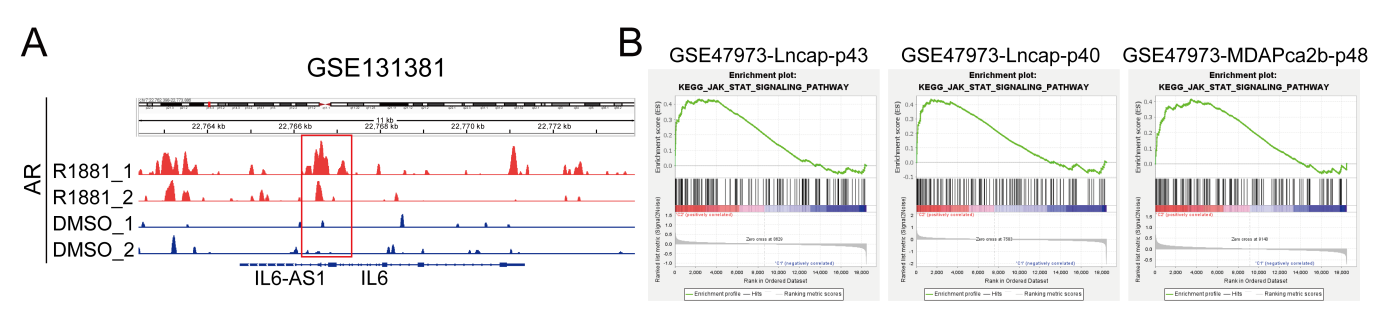


**Figure S5**. AR directly binds to the IL6 locus and activates downstream JAK/STAT signaling.

(A) ChIP-seq signal tracks (GSE131381) demonstrating AR binding enrichment at the IL6 promoter region in THP-1 cells treated with AR agonist R1881 or DMSO. The red box highlights the specific binding peak. (B) GSEA analysis revealing significant enrichment of JAK/STAT signaling pathway upon IL-6 stimulation in the GSE47973 dataset (Lncap-p43, Lncap-p40, MDAPca2b-p48).


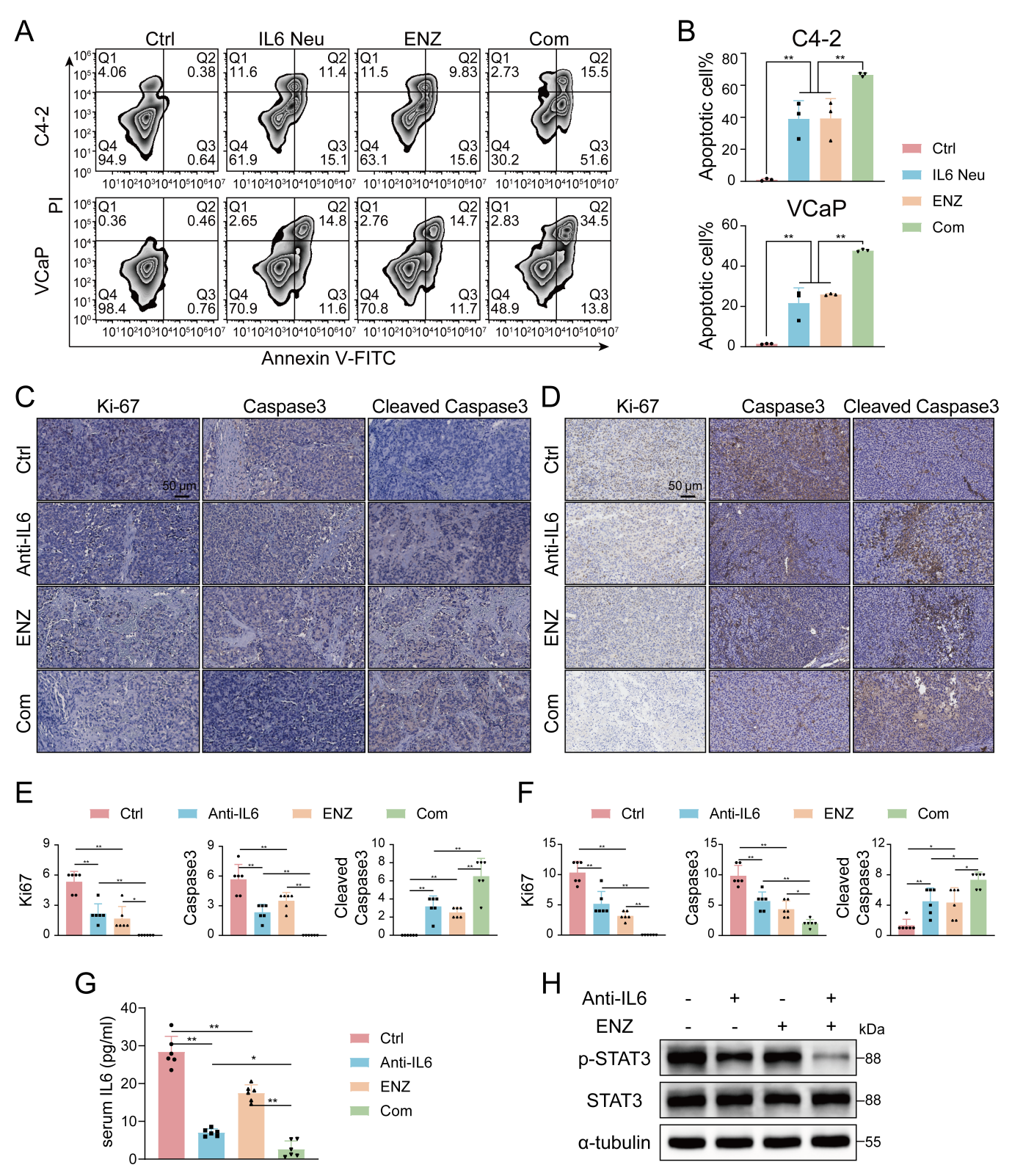


**Figure S6**. IL-6 blockade sensitizes PCa cells to ENZ *in vitro* and *in vivo*.

(A, B) Flow cytometry analysis (A) and quantification (B) of apoptosis in C4-2 and VCaP cells treated with vehicle (Ctrl), IL-6 neutralizing antibody (IL6 Neu), ENZ, or their combination (Com). (C–F) Representative IHC staining (C, D) and corresponding quantitative analyses (E, F) of Ki-67, Caspase-3, and Cleaved Caspase-3 in tumor sections derived from the PDX (C) and orthotopic (D) mouse models. Scale bar = 50um. (G) ELISA quantification of serum IL-6 levels in tumor-bearing mice across the indicated treatment groups. (H) WB analysis of p-STAT3 and STAT3 levels in tumor xenografts.

| Characteristics | HSPC (n=135) | CRPC (n=18) | *p* value |
| --- | --- | --- | --- |
| Age (years) |  |  | 0.315 |
| <70 | 66 (48.9%) | 6 (33.3%) |  |
| ≥70 | 69 (51.1%) | 12 (66.7%) |  |
| Grade |  |  | **0.007** |
| 1 | 10 (7.4%) | 1 (5.6%) |  |
| 2 | 51 (37.8%) | 1 (5.6%) |  |
| 3 | 29 (21.5%) | 2 (11.1%) |  |
| 4 | 21 (15.6%) | 6 (33.3%) |  |
| 5 | 24 (17.8%) | 8 (44.4%) |  |
| T Stage |  |  | **<0.001** |
| T1 | 0 (0.0%) | 0 (0.0%) |  |
| T2 | 74 (54.8%) | 2 (11.1%) |  |
| T3 | 60 (44.4%) | 13 (72.2%) |  |
| T4 | 1 (0.7%) | 3 (16.7%) |  |
| N Stage |  |  | **0.025** |
| N0 | 113 (83.7%) | 12 (66.7%) |  |
| N1 | 15 (11.1%) | 6 (33.3%) |  |
| Nx | 7 (5.2%) | 0 (0.0%) |  |
| M Stage |  |  | **0.022** |
| M0 | 122 (90.4%) | 14 (77.8%) |  |
| M1 | 6 (4.4%) | 4 (22.2%) |  |
| Mx | 7 (5.2%) | 0 (0.0%) |  |
| tPSA (ng/mL) |  |  | **0.008** |
| <20 | 99 (73.3%) | 7 (38.9%) |  |
| ≥20 | 32 (23.7%) | 10 (55.6%) |  |
| Unknown | 4 (3.0%) | 1 (5.6%) |  |
| fPSA (ng/mL) |  |  | **0.038** |
| <10 | 98 (72.6%) | 14 (77.8%) |  |
| ≥10 | 3 (2.2%) | 3 (16.7%) |  |
| Unknown | 34 (25.2%) | 1 (5.6%) |  |

**Table S1**. Baseline characteristics between patients with HSPC and CRPC

Bold values indicate statistical significance (*p*<0.05).

**Table S2**. The shRNA and PCR primers sequences

|  | Gene names | Sequences |
| --- | --- | --- |
| shRNA | m-ANXA2-1 | GTATGATGCTTCGGAACTAAA |
|  | m-ANXA2-2 | CGAGACAAGGTCCTGATTAGA |
|  | h-AR-1 | GGAGCUCUCACAUGUGGAATT |
|  | h-AR-2 | GCAGAAAUGAUUGCACUAUTT |
|  | h-IL6-1 | GAGTACCTCCAGAACAGATTT |
|  | h-IL6-2 | GCAGGACATGACAACTCATCT |
|  | h-IL6-3 | ATGAGCGTTAGGACACTATTT |
| RT-PCR primer | h-AR-Forward | CCAGGGACCATGTTTTGCC |
|  | h-AR-Reverse | CGAAGACGACAAGATGGACAA |
|  | h-IL6-Forward | ACTCACCTCTTCAGAACGAATTG |
|  | h-IL6-Reverse | CCATCTTTGGAAGGTTCAGGTTG |
|  | h-GAPDH | GGAGCGAGATCCCTCCAAAAT |
|  | h-GAPDH | GGCTGTTGTCATACTTCTCATGG |
| ChIP-PCR primer | h-IL6-Forward | GTGGTCGCATGCATCTGTAGTC |
|  | h-IL6-Reverse | GAGAAGGAGTCTTGCTCTGTTG |
|  | m-IL6-Forward | AGATAGCCAAGAGACCACTG |
|  | m-IL6-Reverse | GGAGTTGCCAGGTGGGTAAA |
